# Dyadic Coping in Couples: A Conceptual Integration and a Review of the Empirical Literature

**DOI:** 10.3389/fpsyg.2019.00571

**Published:** 2019-03-26

**Authors:** Mariana Karin Falconier, Rebekka Kuhn

**Affiliations:** ^1^Department of Family Science, School of Public Health, University of Maryland, College Park, MD, United States; ^2^Department of Psychology, University of Zurich, Zurich, Switzerland

**Keywords:** coping, couples, models, review, stress

## Abstract

The present review on dyadic coping (DC) aims at providing a critical integration of both the conceptual and empirical DC literature and overcoming the limitations of past reviews by (a) describing, comparing, and integrating all the DC models, (b) presenting and integrating findings from studies based on DC models, and (c) suggesting directions for further research. The DC models identified and compared include: The congruence model (Revenson, 1994), the relationship-focused model (Coyne and Smith, 1991; O'Brien and DeLongis, 1996), the communal coping model (Lyons et al., 1998), the systemic-transactional model (Bodenmann, 1995, 1997), the relational-cultural model (Kayser et al., 2007), and the developmental-contextual coping model (Berg and Upchurch, 2007). After discussing each DC model, we advance a conceptual integration of all models, which serves as the framework to organize the review of the empirical literature. This integration includes the following DC dimensions: (a) Stress Communication, (b) Positive DC by One Partner (supportive DC, empathic responding, delegated DC, active engagement), (c) Positive Conjoint DC (common, collaborative, communal, mutual responsiveness); (d) Negative DC by One Partner (protective buffering, overprotection, and hostility/ambivalence), and (e) Negative Conjoint DC (common negative DC, disengaged avoidance). Developmental, relational, and contextual variables are included as factors shaping DC. To be included in the empirical review, articles had to be published in or a peer-reviewed journal in English and/or German before 2017 and include an original empirical study guided by one of the DC models. The review included 139 studies and, with the exception of the congruence model whose findings were discussed separately, findings were presented for overall DC and each of the dimensions identified in the conceptual integration. Findings were grouped also according to whether the stressor related or not to a medical or mental health condition. Demographic and cultural factors affecting DC were discussed. Overall, the empirical review suggests that in Western couples, positive individual, and conjoint DC forms, taken together or separately, have individual and relational benefits for couples coping with stress in general and/or mental health or medical stressors. Research on DC can be expanded to include other populations and stressors and use improved designs.

For decades the study of stress and coping strategies focused mainly on the individual, without considering the reciprocal influential processes that are part of relational contexts (e.g., Lazarus and Folkman, 1984). The focus was limited to the stressed individual and the role that partner's support might play in reducing his or her stress. It was only in the last two decades that scholars adopted a more systemic perspective and shifted their view of stressors as affecting only one partner to affecting both, either directly when partners face the same stressful event such as a *dyadic stressor* (e.g., financial problems) or indirectly when the stressor may be initially related to one partner (e.g., a medical problem) but then spills into the relationship and ends up affecting the other partner as well. In other words, stress in couples was no longer conceptualized as an individual phenomenon but as a *dyadic* affair (e.g., Bodenmann, 1995, 1997; Lyons et al., 1998). This dyadic conceptualization of stress emphasizes not only the interdependence of partners' stress experience but it also places the coping process with external stressors (stressful situations originating outside the couple's relationship) in a relational context in which partners respond not only to their individual stress but also to each other's stress. This interpersonal view opens a new understanding of how couples deal with everyday stress as well as critical life events. Partners' coping responses to each other's stress resulting from circumstances outside the relationship is usually referred to as *dyadic coping* (DC). For stress that is experienced as a result of within-the-relationship stressors (e.g., conflict with partner, infidelity), partners usually use their own individual coping strategies and they do not rely on dyadic coping since they are not likely to help each other cope with that type of stress.

In line with this shift toward an interpersonal view of stress and coping in couples, various DC models have been proposed, such as the relationship-focused model (Coyne and Smith, 1991), the congruence model (Revenson, 1994), the systemic transactional model (Bodenmann, 1995, 1997), and the developmental-contextual coping model (Berg and Upchurch, 2007) among others. DC research has been typically guided by one of these conceptual models, each of which may offer some unique DC perspective. However, significant conceptual overlap also exists among those DC models (e.g., Falconier et al., 2015). A critical examination of such models and their research reveals that each model offers a partial view of the DC process and that their studies fail to integrate findings from studies guided by other DC conceptual models that focus on similar DC aspects. This fragmented approach has prevented from capturing the progress made in the field toward understanding the DC process and answering questions such as whether some aspects of DC process are more beneficial than others, whether the DC benefits vary by stressors, whether there are demographic variations in DC, or where further DC research is needed. Therefore, the present manuscript reviews and analyzes the conceptual and empirical literature with the aim of providing an integrated view of the DC process, organizing the accumulated empirical knowledge, and identifying areas for further research. In the first section of the manuscript we describe each DC model and its unique conceptual contributions in understanding the DC process while identifying its conceptual overlap with other DC models. This comparative analysis leads to the proposal of an integrative model that includes all the different dimensions of DC identified across different models while eliminating unnecessary conceptual overlaps. The resulting integrative model allows to present a comprehensive approach to understanding the DC process and guides the organization and presentation of the accumulated empirical findings in the DC field in the second section of the present manuscript. After describing the methodology used to conduct the empirical review, this second section reports research findings in each of the DC dimensions included in the integrative model, while differentiating findings regarding medical and mental health stressors vs. other types of (non-medical) stressors. The discussion of such findings illuminates areas for further research. In other words, the goals of this paper are: (a) to describe each DC model, its contributions to understanding the DC process, and its conceptual differences and overlaps with other models; (b) to develop a model that includes all dimensions and factors identified in each DC model in order to have a comprehensive understanding of the DC process and allows to organize the empirical literature; (c) to summarize the findings from the empirical literature regarding each DC dimension and the potential effects of demographic (age, gender) and contextual factors (e.g., culture) on couples coping with medical and non-medical stressors, and (d) to discuss further research directions in the field. To the best of our knowledge, this is the first manuscript that attempts to provide such an integration and review of all the DC conceptual and empirical literature. Previous attempts to review and/or conceptually integrate the DC literature have focused exclusively on one stressor, mostly couples coping with cancer (Traa et al., 2015), have reviewed only the association between DC and relationship satisfaction (Falconier et al., 2015), or have included mostly studies applying only one DC model (Staff et al., 2017). These reviews have failed to integrate conceptually all DC existing models and/or have left out a large number of the studies that have been published in the last two decades.

## Dyadic Coping Models

The initial DC models were formalized in the 1990s and each of them followed Lazarus and Folkman's conceptualization of stress as resulting from the perception or appraisal that the demands of a situation exceed the resources available to deal with such demands. These initial models were the congruence model (CM; Revenson, 1994), the relationship-focused model (RFM; DeLongis and O'Brien, 1990; Coyne and Smith, 1991), the communal coping model (CCM; Lyons et al., 1998), and the systemic-transactional model (STM; Bodenmann, 1995; Bodenmann et al., 2016). In the last decade these initial models were expanded to incorporate developmental and cultural aspects resulting in the relational-cultural coping model (RCCM; Kayser et al., 2007; Kayser and Revenson, 2016), and the developmental-contextual coping model (DCCM; Berg and Upchurch, 2007). The following section includes a description of each DC model, the research areas in which each model has been applied and the instruments that have been used to measure the constructs. The presentation of each model seeks to uncover the unique contributions of each model to the understanding of the DC process while identifying conceptual overlaps with other DC models. This comparative analysis is necessary to create a theoretical framework that integrates all the conceptual developments about the DC process in the field.

### The Congruence Model (CM)

During the 1980s different researchers became interested in the interplay between partners' individual coping styles by examining the effect of similarities and dissimilarities between those coping styles on individual and relational outcomes (e.g., Barbarin et al., 1985). Their studies marked the beginning of the DC field by considering one partner's stress and coping in relation to the other partner's and therefore acknowledging the interpersonal context of the stress and coping process in couples. For example, Cronkite and Moos (1984) studied whether similarity between partners' coping styles alleviated the effects of illness-related stress and concluded that “the personal coping resources and coping responses of each partner can alter the impact of stress and the effectiveness of coping” (p. 389). Later on, Revenson (1994) moved beyond the similarity or dissimilarity between partners' coping strategies and instead focused on the congruence, or *fit*, between the partners' coping styles, that is, the degree to which partners' coping responses are coordinated and mutually supporting. Revenson coined the term “congruence” coping and advanced the idea that the coordination of coping efforts or mutually reinforcing coping strategies can lead to positive psychosocial outcomes.

The CM and the dissimilarity/similarity framework has been mostly applied to the study of couples coping with stress in general (Cronkite and Moos, 1984; Giunta and Compas, 1993), cancer (e.g., Kraemer et al., 2011), and multiple sclerosis (Pakenham, 1998). Those studies usually assess each partner's individual coping style through well-known individual coping measures such as the Revised Ways of Coping Scale (Vitaliano et al., 1985) or the Coping Strategies Inventory (Carver et al., 1989).

Unlike any other DC model, the CM focuses on the interplay between partners' individual strategies to cope with their own stress rather than on partners' conjoint strategies to cope with common stressors or a partner's coping responses to the other partner's stress. In this regard it is the only DC model that examines the interpersonal effects of individual coping strategies on couple functioning.

### The Relationship-Focused Model (RFM)

There were two groups of researchers, one led Coyne and Smith (1991) and another led by DeLongis and O'Brien (1990), that were the first to consider that in addition to individual emotion- and problem-focused strategies to cope with stress, individuals also responded with *relationship-focused* strategies “aimed at managing, regulating, or preserving relationships during stressful periods…particularly when stressors occur in interpersonal contexts” (O'Brien and DeLongis, 1996, p. 782). Each of these groups of scholars focused on different dimensions of the RFM, but both groups attended not to what each partner did to manage their own stress but to what each partner did to help the other partner cope with a stressful situation. Coyne and Smith (1991) studied the way couples responded to a partner's myocardial infarction and identified two coping mechanisms that had relationship-focused function: *active engagement* and *protective buffering*. During active engagement, an individual provides support to the sick partner by involving him or her in conversations about how she or he is thinking and feeling, or about other issues around the medical condition. As such, active engagement would be expected to represent a positive form of support and be related to positive outcomes for the stressed individual and their relationship. Protective buffering refers to the partner's efforts to hide or deny concerns and worries and yielding to the other partner to minimize conflict. Even though this form of coping may be triggered by a positive intent, most studies have shown that it usually has a negative impact on the stressed individual and the couple's relationship (for a review see Falconier et al., 2015). In collaboration with Fiske, Coyne and Smith (1991) also identified another RFM coping strategy: *overprotection*. This coping form is seen when a partner underestimates the sick individual's capabilities and therefore, he or she provides unnecessary support (practical or emotional) or restricts the sick partner's activities. Overprotection can be viewed conceptually as a negative form of dyadic coping and empirical evidence has provided support for its detrimental impact at the individual and relational levels (for a review see Falconier et al., 2015).

Coyne and Smith's model has been mostly applied to the study of couples coping with medical condition such as cancer (e.g., Hinnen et al., 2008), diabetes (e.g., Schokker et al., 2010), Alzheimers (Kramer, 1993), chronic-obstructive pulmonary disease (COPD; Snippe et al., 2012), or smoking (Butler et al., 2014). In order to measure RFM strategies, Coyne and Smith (1991) developed a self-report instrument known as the *Relationship-Focused Coping Scales* with subscales that assess active engagement and protective buffering, and in collaboration with Fiske et al. (1991) they developed a scale for overprotection.

Instead of defining three different specific dimensions, DeLongis and O'Brien (1990) distinguished between positive and negative RFM strategies. Positive strategies included empathy, providing support, and compromise, similar to the STM's supportive DC, whereas negative strategies included withdrawal and hostility, similar conceptually to the ambivalent/hostile negative DC from STM. Over time O'Brien et al. (2009) focused particularly on the use of one form of positive relationship-focused coping: *empathic responding*. This DC form involves “the non-stressed partner's efforts to view the world from the other partner's perspective, experience the affective and cognitive associations that the stressful situation is evoking for the other partner, understand the partner's psychological states in his or her communication” (O'Brien et al., 2009, p. 783). Studies examining empathic responding have focused on stepfamilies (e.g., Lee-Baggley et al., 2005) and medical stressors (e.g., Marin et al., 2007) and have used daily process methods such as structured diaries and the *Empathic Responding Scale* (O'Brien and DeLongis, 1996).

Unlike the CM, the RFM shifted the attention away from what partners do to cope with their own stress to identify what successful and unsuccessful strategies a partner uses to help the other partner cope with his or her own stress. In doing so, the RFM has uniquely contributed to our understanding of DC dimensions by describing protective buffering and overprotection as individual mechanisms that people tend to use to help their romantic partners cope with stressful situations, particularly medical conditions, but that they may end up having a negative impact. Similarly, another unique contribution lies in the identification of active engagement in helping a partner express his or her thoughts and feelings about a medical condition as a strategy to help a partner cope with a stressful health issue. By contrast, empathic responding, which is the other positive DC dimension described by the RFM, bears similarities with the STM construct of emotion-focused support provided by a partner to the other to help him or her cope with stress. Despite its unique contributions, the RFM does not include what partners do conjointly to cope with stress and acknowledge the role of contextual factors (e.g., culture) in shaping how couples cope with stress.

### The Communal Coping Model (CCM)

In 1998 Lyons and colleagues introduced the term *communal coping* as occurring “when one or more individuals perceive a stressor as ‘our’ problem (a social appraisal) vs. ‘my’ or ‘your’ problem (an individualistic appraisal) and activate a process of shared collaborative coping” (p. 583). They viewed communal coping as a process happening in families and communities and that could have benefits for relationships and for the individual. According to the CCM there are three components involved in communal coping. First, at least one of the individuals in the relationship must have a *communal coping orientation*, that is, believe that conjoint coping is beneficial, necessary and/or expected to deal with a problem. Second, the process of communal coping requires *communication about the stressor*, that is, individuals must share the details and meaning of the situation. Third, individuals respond to the stressor with *cooperative action*, that is, they collaborate to develop strategies that reduce the negative impact of the situation and address the demands of the stressful situation.

Even though the CCM does not apply to couples coping only, various scholars have argued that it is a good model to understand couples coping with medical stressors. Lewis et al. (2006) have argued that couples' communal coping can lead to the adoption of risk-reducing health habits while Helgeson et al. (2017) have proposed that the CCM can be used to explain the “optimal pathway to patient adjustment among couples in which one person faces a chronic illness” (p. 1). Helgeson and colleagues emphasize that the primary goal of communal coping is to enhance not the relationship but the patient's adjustment to chronic illness. Unlike Lyons' formulation of communal coping, Helgeson and colleagues considered that shared illness appraisals may lead not only to collaboration but also to support interactions.

When communal coping has been studied in the context of couples' DC, it has focused mostly on coping with one partner's medical conditions and it was measured mostly through linguistic markers. Such studies (e.g., Rohrbaugh et al., 2012) have typically used the Linguistic Inquiry Word Count program (Pennebaker et al., 2007) to count partners‘use of first-person plural pronouns in couple conversations (e.g., transcripts of marital interaction tasks or intervention sessions) such as *we, us*, or *our*, also referred to as *we*-talk. Some studies (e.g., Rohrbaugh et al., 2008) have also used two self-report questions, one asking the extent to which a partner views the other partner's medical condition as “our problem” and another question inquiring about the extent to which both partners work together to resolve that problem.

The CCM has emphasized the benefits of perceiving, communicating, and coping with a partner's medical condition not as an individual issue but as a couple's problem. This emphasis is also part of the other models such as the RCCM, the DCCM, and the STM that have also highlighted the benefits of such a communal approach to problems that have long been perceived and dealt with from an individual perspective. Similar to the RCCM, the CCM has to be credited for its examination of stress appraisals as communal. Unlike the DCCM and the STM, which have focused on measuring communal or collaborating coping strategies, the CCM has studied the extent to which couples appraise individual stressors such as a medical condition as a shared problem or “our” problem. Nonetheless, compared to other DC models, the CCM has had a narrower focus for its almost exclusive interest in medical problems or individual stressors. As it will be discussed later, models such as the STM include conjoint or collaborative coping as a strategy that couples may use to deal not just with individual stressors that may affect both partners but also with common or dyadic stressors. In addition, the CCM has not included other DC processes in the context of couples coping with stress such as when one partner offers emotion- or problem-focused support to a stressful partner and the stressor is not perceived as “our” problem.

### The Systemic-Transactional Model (STM)

Unlike the RFM or the CM that originated in the study of couples where one partner had a serious medical condition, the STM (Bodenmann, 1995) focused on examining coping processes in couples dealing with daily hassles or minor chronic stressors. According to the STM, when partners experience stress, they resort to individual and dyadic coping strategies as well as seeking support outside the couple's relationship and “dyadic coping is used most often after individual coping efforts have been made and failed” (pp. 36–37). Similar to the CCM, the STM also includes stress communication as part of the dyadic coping process. According to the STM, each partner communicates his/her experience of stress to the other partner either verbally, non-verbally, and/or para-verbally and the other partner perceives, interprets, and decodes these signals and responds to the stressed partner with some form of coping “to maintain or restore a state of homeostasis as individuals, as a couple, and with regard to other people in the couple's social world” (Bodenmann, 2005, p. 36). The couple's coping process is seen as being affected by various factors such as context, type of stressor, degree of concern for both partners, attributions of causes of the stress, personal, motivational, and relational factors.

The STM is a comprehensive DC model as it involves various dimensions of positive and negative DC. Positive DC forms are viewed as benefiting both partners and their relationship and include *supportive, delegated*, and *common DC*. Supportive DC refers to one partner's attempts to assist the other partner in his/her coping efforts through problem-focused (e.g., giving advice or helping to find solutions) or emotion-focused strategies (e.g., showing understanding). Delegated DC involves efforts to help the partner reduce the stress by taking over some of his/her responsibilities. Common DC refers to coping strategies in which both partners participate more or less symmetrically or complementarily and can be either problem-focused (e.g., finding a solution together) or emotion-focused (e.g., emotion-regulating together). Similar to CCM, common DC is likely to occur in situations that are affecting both partners and that are considered dyadic stressors or “we-experiences” (e.g., birth or death of a child, economic problems, child behavior problems, etc.), but unlike the CCM, it is also considered a coping strategy that may also happen in response to situations that may be initially related to one partner (e.g., job loss, disease) but is experienced as a situation affecting both partners and therefore as “we-stress” or “we-disease” (Bodenmann et al., 2016).

STM negative DC forms include *hostile, ambivalent*, and *superficial* efforts to assist the stressed partner. Hostile DC involves distancing, mocking, showing disinterest, or minimizing the seriousness of the situation. Ambivalent DC refers to offer support unwillingly or showing that support should not be necessary. Superficial DC refers to insincere efforts to support the stressed partner. Badr et al. (2010) have also added a negative form of common DC that is characterized by mutual avoidance or withdrawal.

The STM has been applied in studies of couples coping with a medical illness such as cancer (see Traa et al., 2015 for a systematic review) or COPD (Meier et al., 2012), but also with other non-medical stressors such as depression (e.g., Bodenmann et al., 2001), post-traumatic stress disorder (Witkovsky and Braakmann, 2015), immigration issues (Falconier et al., 2013a), coping with the death of a child (Bergstraesser et al., 2015), or even general stress (Rusu et al., 2016). All STM studies used the instrument developed by Bodenmann (2008) to assess DC: the Dyadic Coping Inventory (DCI). This scale was initially made up of 55 items but it has been further developed into the most common 37-item version. The DCI has been used in at least 35 countries (Hilpert et al., 2016), and validated for over 10 different cultural groups (for a review see Falconier et al., 2015). A standardized coding scheme based on the STM concepts has also been developed for observations of couples' conversations (Bodenmann, 2000).

Even though the STM did not explicitly incorporate any cultural factors in its original formulations, it did acknowledge that contextual factors could affect the DC process. Furthermore, more recently Falconier et al. (2016a) included culture into the STM as a powerful contextual factor that may affect “whether and to what extent situations are considered stressful, and whether the stressor is viewed as concerning only one partner (individual stressor) or both partners (common stressor)” (p. 28). Cultural factors “may affect the extent to which couples prefer dyadic coping over other coping strategies, the potential benefits of dyadic coping over other coping mechanisms” and shape “the specific factors that favor dyadic coping, and the preference for relying more on some dyadic coping dimensions over others” (Falconier et al., 2016b, p. 304). Falconier et al. (2016b) identified the culture's communication style and individualistic vs. collectivistic orientation as factors that may influence stress appraisal and coping responses.

The STM is the model that has guided most of the research in the DC field (Falconier et al., 2016a). This may be due to the fact that the STM is the model that includes most DC dimensions. Whereas, the RCFM has focused on what one partner does to assist the other partner cope with his or her stress and the CCM has been concerned with the appraisal of the stressor as a “we” problem and collaborative coping strategies, the STM has provided a broadened framework in which stress appraisal (“our” problem vs. “your” or “my” problem” is included and both, collaborative and individual mechanisms for assisting one partner to cope with stress or for partners coping with stress together are present. In addition to its comprehensiveness, the STM has been the only one to emphasize the stress communication process as a DC dimension.

Nonetheless, despite its comprehensiveness and broad appeal, STM studies have relied mostly on the DCI, which measures only coping strategies and not stress appraisal. In contrast to the CCM, the STM has not produced studies examining its conceptualizations on stress appraisal and its link to coping strategies. The STM does not include either particular forms of partners' negative or positive support that have been identified in other DC models such as overprotection, protective buffering, or active engagement.

### The Relational-Cultural Coping Model (RCCM)

In applying the STM to the study of couples' coping with cancer, Kayser and Revenson (2016) focused not only on couples' coping strategies but also on the factors that shaped those behaviors. As a result, they developed the relational-cultural coping model (RCCM) which expanded the STM by adding relational and cultural components. First, in terms of relational aspects, Kayser and colleagues found experiencing cancer as “we-stress” or as an individual stressor affecting each partner individually determined whether the couple displayed *mutual responsiveness* or *disengaged avoidance*. Similar to communal coping and the STM common DC, mutual responsiveness, which was associated with experiencing cancer as “we-stress,” referred to coping in which partners communicated about the stress and handled the situation in a coordinated way with both emotion- and problem-focused responses, whereas disengaged avoidant couples, associated with experiencing cancer as an individual problem, described a response in which partners avoided talking to each other and just focused on the practical aspects of coping with the illness. They identified three key relationship factors contributing to mutual responsiveness: *relationship awareness, authenticity*, and *mutuality* (Kayser et al., 2007). Relationship awareness refers to “thinking about the impact of the disease on each partner and the relationship and how to sustain one's relationship given the extra demands of the illness” (p. 415). Authenticity involves “the disclosing of genuine feelings and not hiding them” (p. 416), whereas mutuality refers to “empathy as a way of relating in which each of the partners is participating as fully as possible in a shared experience” (p. 416).

Regarding the cultural component, Kayser et al. (2007) first expanded the STM by acknowledging the role of culture in shaping the way in which couples adapt to stressful situations, but later on, after conducting a study with American, Chinese, and Indian couples (2014), they proposed four specific cultural dimensions that could influence coping: family boundaries (from open to closed), gender roles (from differentiated to flexible), personal control (from acceptance to mastery), and independence (from dependence to independence). Where couples lie on the continuum of each of these dimensions is likely to affect the way in which they cope with cancer and stress in general. The RCCM was developed from qualitative studies and no instrument has been developed to measure its constructs.

The RCCM's identification of mutual responsiveness as involving both “we-ness” stress appraisals and collaborative coping responses and as beneficial in couples' coping with medical conditions is aligned with STM, and particularly, CMM conceptualizations. However, RCCM's identification of key relationship factors that contribute to either mutual responsiveness or disengaged avoidance is a unique contribution. In addition and most importantly, unlike STM and DCCM, the RCCM is the first DC model that attempted to identify through research the cultural aspects that affect stress and coping processes in the couple's context. Furthermore, it is the only model that has produced a study that did not only include a multicultural sample but it actually focused on examining cultural factors to explain differences in that diverse samples. Given these contributions, the RCCM can be characterized as a DC model that focused more on the factors that shape the stress appraisal and coping process rather than on the actual stress and coping mechanisms in couples. Nonetheless, despite its contributions, many of the RCCM concepts still need to be operationalized into measurable constructs that can be used in research. Also, considering its focus on medical conditions, it is unknown whether the cultural and relational factors identified by the RCCM as shaping the stress and coping process apply to other stressful situations.

### The Developmental-Contextual Coping Model (DCCM)

The DCCM was developed by Berg and Upchurch (2007) to understand the process through which couples cope with chronic illness. Similar to the STM, the CCM, and the RCCM, the DCCM highlights the importance of the stress appraisal process that comes prior to the actual coping strategies. Appraisals can be made about the illness controllability and the illness ownership. Similar to other DC models, stress can be perceived as individual (one's own stress), indirectly (through my partner's stress experience), or shared (both partners appraise the stressor as a common one), similar to the “we-appraisal” described by the STM, the CCM, and the RCCM. However, the DCCM also acknowledges that the coping strategy also affects the appraisal processes (e.g., collaborating as one coping strategy activates the belief that the stressor is appraised as a joint stressor).

Unlike other DC models, the DCCM does not focus on stress communication but on the partner's responses, which are viewed on a continuum that spans from under-involvement to over-involvement. The DCCM also argues that DC dimensions identified in other models are one of the four coping strategies on that continuum: uninvolved, supportive, collaborative, and controlling. Uninvolved coping refers to the perception that one's partner is providing no support to help the other cope with stress, whereas supportive coping refers to the perception that the partner is providing such support either emotionally and/or instrumentally. Similar to the STM common DC, the CMM, and the RCCM mutual responsiveness, collaborative coping describes both partners' actions to cope with the stressful situation together. Controlling coping describes moments in which the non-stressed partner “dominates the actions of the other spouse by taking charge and telling the other person what to do” (Berg and Upchurch, 2007, pp. 932–933) and is associated with the protective buffering and overprotection strategies identified in the RFM.

Unlike other models, as the name suggests, the DCCM emphasizes the role of developmental and contextual factors in the appraisal of the stressor and coping responses. In terms of developmental aspects the DCCM argues that coping with an illness varies over time depending on the stage of illness and the life cycle stage. Regarding contextual factors, the DCCM views cultural differences, gender differences, the quality of the couple relationship, and the illness type as affecting stress appraisal and partners' coping responses. Interactions between different factors are taken into consideration so that, for example, different cultural groups experience different diseases at different rates.

The DCCM studies have measured coping through diaries (Berg et al., 2008), the Perceptions of Collaboration Questionnaire (PCQ; Berg et al., 2008), and structured stress and coping interviews (Berg et al., 2008). Even though the model includes developmental and contextual aspects, the DCCM studies have focused only on coping dimensions and demographic variables such as age, gender, and length of relationship.

Compared to other models and similar to the STM, the DCCM offers a more comprehensive framework by including stress appraisal, a partner's both positive and negative responses to the stressed partner, partners' collaborative coping efforts, and contextual factors that can affect stress appraisal and coping processes with medical conditions. However, the model's unique contribution is its proposal that the couple's coping strategies may vary depending on the stage of the illness. In this regard, it is the only DC model to suggest that couples may cope differently over time, even when dealing with the same stressor. This dynamic view of coping seems to be absent in other approaches or studies. Despite these contributions, the DCCM does not include all the DC dimensions identified in other models and that could be relevant to cope with non-medical stressors (e.g., the STM's negative DC).

### Model Integration

**Figure 1:**
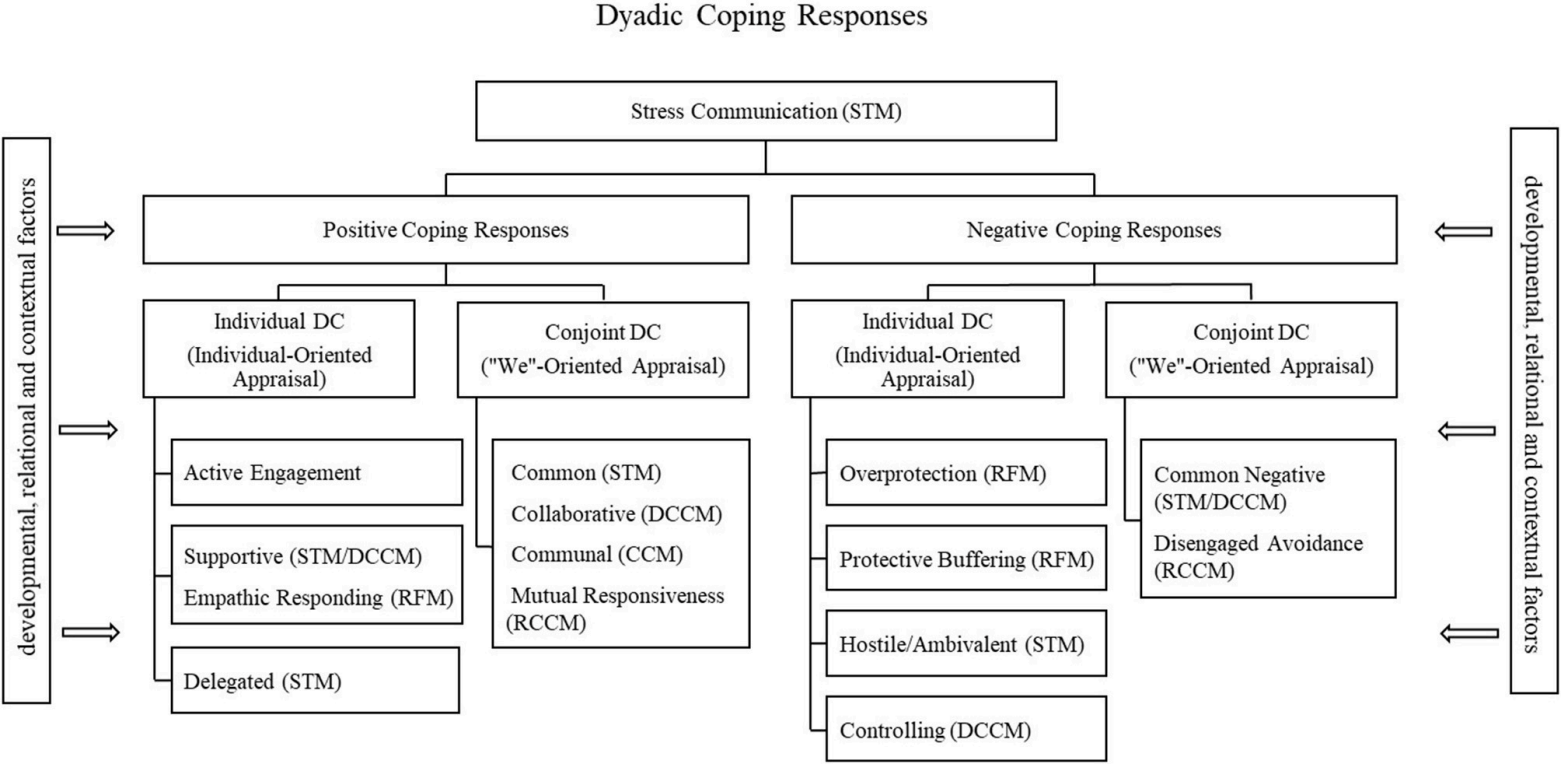
Integration model chart.

When looking at the coping process in the context of couple's relationships, there have been two different approaches. One approach, exemplified in the CM, continues with the tradition of examining the individual coping responses to one's stress, but it brings attention to the relational context by (a) focusing on whether partners' individual coping responses are mutually supportive and (b) whether the interplay of such strategies is beneficial for each partner and their relationship. The other approach, which is represented in the other models and could be viewed as a more dyadic conceptualization, moves beyond individual strategies for coping with one's own stress to focus on what partners do or don't do for each other and together to handle stress. These models typically assume that that when one partner experiences stress, so does the other partner due to the common nature of the stressor or to crossover effects. As discussed in the description of those DC models (the RFM, the CCM, the STM, the RCCM, and the DCCM), each of them seemed to have examined different or overlapping dimensions of the same DC phenomenon and therefore, they could be integrated into a comprehensive DC model. Some of this integration has been attempted before (e.g., Falconier et al., 2015) but, as noted earlier, it has left out some of the DC models or specific dimensions highlighted by each model. After examining conceptual overlaps and differences in the previous sections, our integrated view proposes that the DC process involves partners' communication about their stress and their responses which can be positive or negative and include individual responses to a partner's stress when the stressor is viewed as concerning one partner (individual-oriented appraisal) or conjoint responses when the stressor concerns both partners or an individual stressor is viewed as a “we” or shared problem (“we” oriented appraisal; see Figure 1). Therefore, the integration includes: (a) *Stress Communication* to refer to communication of the experience of stress between partners, (b) *Individual Positive DC* to refer to one partner's positive responses to help the other cope with stress (supportive DC, empathic responding, delegated DC, active engagement), (c) *Positive Conjoint DC* to refer to what partners do together to cope with shared or dyadic stress (common, collaborative, communal DC, mutual responsiveness); (d) *Negative Individual DC* to refer to one partner's negative responses to the other partner's stress (e.g., protective buffering, overprotection, hostile/ambivalent DC, and controlling DC), and (e) *Negative Conjoint DC* to refer to partners' conjoint negative response to deal with a shared or dyadic stress (common negative DC, disengaged avoidance). Similarly to DCCM formulations, developmental, relational, and contextual variables are included in the model as factors that can shape the stress and coping process. The inclusion of the developmental factors indicate that changes in stress appraisal and use of DC strategies may change over time due to the changes or development of the stressful situation. In other words, changes in the stressor may lead to the adoption of different coping mechanisms. Similarly, DC strategies that may have been adopted to first cope with a stressor may be changed for others after some time. For example, a partner may initially respond to her husband's extended family problems with supportive DC but over time she may appraise the situation as a “we” problem and engage in positive conjoint DC. Relationship variables are those characteristics of the relationship that influence the stress appraisal and coping process. The RCCM has already proposed some relationship characteristics such as relationship awareness, authenticity, and mutuality that increase the likelihood that partners will appraise problems as shared and will engage in collaborative forms of coping. It is also possible that other relationship characteristics such as level of intimacy, satisfaction, and ability to resolve conflict constructively also affect the stress appraisal and coping process. Contextual factors refer to socio-economic conditions that may affect the availability of resources (e.g., unemployment, income level), cultural values (e.g., collectivistic vs. individualistic), and/or religious beliefs that may affect stress appraisal and coping in couples.

It is important to note that it is only when a review and analysis of all DC models is made that the uniqueness of the DC concept is fully understood and differentiated from other constructs such as partner's social support. Unlike DC, which is one partner's or both partners' response to the stress experienced by one or both partners, social support by a partner is not necessarily provided to assist the partner cope with stress. Social support, which can be informational (e.g., recommendations, advice, helpful information), instrumental (e.g., financial, material, or physical assistance), emotional (e.g., expression of affection, caring), and/or companion (availability of partner) can also occur in the absence of a stressor (Kent de Grey et al., 2018).

## Methodology for the Review of the Empirical Literature

### Literature Search

In order to conduct the review of all the empirical literature guided by the DC models discussed above we conducted the search in the *Psychology and Behavioral Sciences Collection* from *EBSCOhost* and in Psych-INFO in 2017 and 2018. We used the following inclusion criteria for selecting studies: (a) be published in or before 2017, (b) include an original empirical study guided by one of the DC models identified in the present review, and (c) be published in a peer-reviewed journal in English and/or German in order to guarantee the scientific merit. All DC models but the STM were developed in English speaking countries, whereas the STM was developed in Switzerland. Therefore, we included journal articles both in English and German to increase the likelihood of including as many studies as possible for each DC model. In addition, the search included terms related to the models' names and constructs such as “stress,” “couple,” “relationship,” and “intimate.”

### Study Selection

Studies were selected when they mentioned and based themselves explicitly on the specific model or when they used one of the model-related questionnaires developed by the authors of the models. Articles were excluded, for example, if they focused on relationship-internal conflicts instead of a relation-ship external stressor, or if they did not include any coping efforts by any partner.

**Figure 2:**
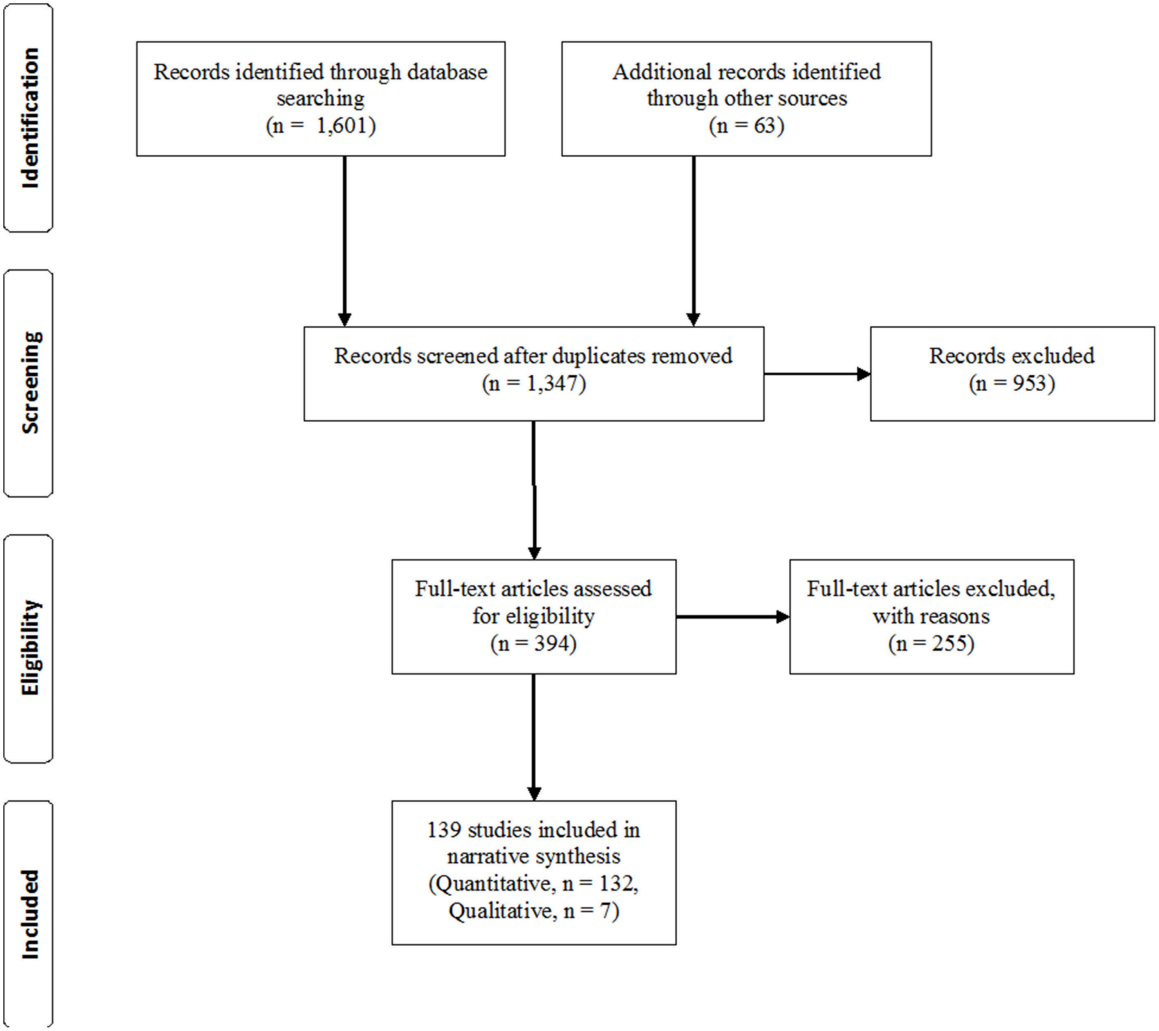
Prisma flowchart.

The initial search through these databases yielded 1,601 results and 63 more articles were added after inspecting reference lists of included articles or because the model developers, when consulted, identified additional articles that our database search had failed to identify (see Figure 2). We removed 317 duplicate articles and screened 1,347 records in a two-step process (abstract screening, full-text screening).

The coding team included the present authors, graduate assistants, and alumni from two different universities. Coders read abstracts of all 1,347 articles and eliminated 953 records not meeting eligibility criteria. We read 394 records in full and 255 articles were further excluded for not meeting eligibility criteria (e.g., examination of only one partner's individual coping strategies, not being an empirical article, not applying a DC model, or not focusing on stress). When in doubt or disagreement, coders consulted with the rest of the coding team until an agreement was reached. To ensure accuracy, both coders read and agreed on 43% of the final articles. The current review ended up including 132 quantitative studies and 7 qualitative studies (see Figure 2 and summary of studies table available online as Supplementary Material). In total, over 37,000 couples and individuals participated in the different studies (range: 10–7,973 individual and couples). Studies were mainly cross-sectional (66%: 92 out of 139) and 47 were longitudinal. In addition, seven studies reported an experimental design (e.g., stress tests with experimental groups).

### Data Extraction

Each study was entered into a database identifying the authors, title, sample, DC model, DC measure, non-DC measures, study design, and main findings. Articles were classified into the different DC models either because they made explicit the model that guided their research or because they used DC constructs or measurements developed after the DC models. The classification resulted in the following number of articles for each model: CM: 10; RFM: 34; STM: 78; CCM: 7; RCCM: 2; DCCM: 8 (see summary of studies in Supplemental Material).

## Findings From the Empirical Literature

The review of the empirical literature has been organized into two parts. The first part presents findings from studies examining the interplay of partners' individual coping styles, and therefore mostly related to the CM. The second part discusses findings from the rest of the studies on DC that met the inclusion criteria for the present review. Findings in this second part are presented for DC as an overall construct first. Then, with the exception of negative conjoint responses and controlling DC for which no studies were found, for each of the DC dimensions outlined in the integrative model: Stress communication, individual (active engagement, supportive DC, empathic responding, and delegated DC), and conjoint (common DC/collaborative DC/communal DC/mutual responsiveness) positive coping responses, and individual (overprotection, protective buffering, and hostile/ambivalent DC) negative coping responses. In this way studies guided by different DC models but that tap on the same DC dimension can be presented together. For example, findings on STM's common DC, DCCM's collaborative DC, and CCM's communal coping can be discussed together, obtaining a full picture on the accumulated knowledge in the field regarding couples' conjoint strategies to cope with stress. In addition, findings on couples coping with medical or mental health stressors have been separated from those on couples coping with other types of stressors, referred to as non-medical stressors. The review also includes a final section that discusses the demographic and contextual/cultural differences identified for overall DC and each dimension.

## Congruence Between Partners' Individual Coping

Except for two studies (Cronkite and Moos, 1984; Bodenmann et al., 2011), research on similarity between partners' individual coping strategies have been all related to medical stressors. Overall, findings suggest that positive individual and relational outcomes in stressful situations are not necessarily the result of similarity between partners' individual coping strategies. For example, partners' similarity in emotional and problem-focused coping helped women with non-metastatic cancer adapt 10 months later, but it was dissimilarity in emotional coping that predicted women to be happier with their couple's relationship (Kraemer et al., 2011). Israeli partners' similarity in monitoring as an information seeking style predicted better adjustment in women with cancer; however, similarity in blunting as an information seeking style predicted better adjustment in men with cancer but predicted psychological distress in their caregivers (Barnoy et al., 2006). Similarly, a study on Australian couples with MS (Pakenham, 1998) found dissimilarity in problem-focused coping to be associated with lower collective depression and better individual adjustment in both partners. In a study of parents of children diagnosed with cancer, however, similarity in emotion-focused coping helped parents be more optimistic, but it was the complementarity in problem-focused coping that predicted better marital quality and support (Barbarin et al., 1985).

Even when similar stressors are considered, results have not been consistent for similarity in coping styles. On the one hand, similarity in emotion-focused coping predicted better adjustment in women with breast cancer in Kraemer et al. study (2011) but it did not in a study by Ben-Zur et al. (2001) in which women with breast cancer reported more psychological distress and poorer functioning when both partners relied on emotion-focused coping. Nonetheless, this study also showed that complementarity in emotion-focused coping and that women's avoidance and men's preference for problem solving also predicted women's depression. Similarly, women's avoidance and men's problem-focused had been found in an earlier study to be associated with women's depression and men's physical symptoms in a sample of urban couples (Cronkite and Moos, 1984). However, in that study men were also more depressed when both partners used avoidance coping strategies. But again, by contrast, two American studies (Giunta and Compas, 1993; Fagundes et al., 2012) found that similarity in avoidance did not predict negative affect or psychological distress.

In short, studies on similarities between partners' individual coping styles offer inconsistent findings, even when focusing the same stressor. No socio-demographic, developmental, or cultural factors explained the different results either, all of which lends support to the idea that partners' individual coping styles should be examined in terms of the extent to which each partner's style supports the other partner's instead of blocking them or even creating another source of stress.

## Overall DC (STM)

Several studies within the STM framework have examined DC overall as the aggregation of all or some of the DC dimensions. Furthermore, some of these studies have specifically focused on overall positive DC, excluding negative DC forms.

### Coping With Non-medical Stressors

At the individual level DC has been related to positive individual forms of coping (e.g., Bodenmann et al., 2010a); less neuroticism (Merz et al., 2014), more daily physical activity in women (Reed et al., 2016), higher life satisfaction (Gabriel et al., 2016), and lower anxiety, insomnia, social dysfunction, and depression in women (Bodenmann et al., 2011). Furthermore, in experimentally induced stress conditions, DC was found to reduce stress levels (Meuwly et al., 2012) and low DC was associated with immune reactivity (Reed et al., 2016).

In terms of benefits for the relationship, DC has been associated with tenderness and togetherness (Bodenmann et al., 2006), higher sexual satisfaction, sexual behaviors, and orgasms in women (Bodenmann et al., 2010a), and relationship satisfaction and constructive communication in Western including American couples (e.g., Randall et al., 2015), Latino (e.g., Falconier et al., 2013b), and European couples (e.g., Vedes et al., 2013; Zeidner et al., 2013). Longitudinal studies in Swiss couples have found that DC predicts men's relationship satisfaction 10 years later (Ruffieux et al., 2014) and that couples maintain their relationship satisfaction over a 5 year-period if they are highly involved in DC, but their relationship satisfaction declines if they are not high on DC (Bodenmann, 2000). Some findings even suggest that DC is a better predictor of relationship satisfaction than individual coping (Papp and Witt, 2010) and may be beneficial above and beyond positive communication (Nussbeck et al., 2012). According to a large study across 35 different nations that included not only Western but also African and Asian countries, the extent to which one partner perceives the other as providing DC plays a more important role in predicting relationship satisfaction than the actual extent to which one partner reports engaging in DC (Hilpert et al., 2016). Furthermore, perceived similarity in DC between partners matters more for relationship satisfaction than the actual similarity (e.g., Iafrate et al., 2012). DC can also serve as a predictor for relationship stability. After 5 years, couples could be correctly classified in 73% of the cases regarding whether they would separate or stay together according to their level of DC (Bodenmann and Cina, 2005). In addition, DC has been found to attenuate the negative impact of chronic external stress on chronic internal stress (spillover), particularly for women (Merz et al., 2014), and relationship stability (e.g., Bodenmann and Cina, 1999). Positive DC has also been found to moderate the effects of stress on verbal aggression and anger (Bodenmann et al., 2010b).

Some factors affect partners' likelihood to become involved in DC. Stressors external to the couple's relationship decreases partners' use of DC strategies (e.g., Gabriel and Bodenmann, 2006a) but dyadic empathy (Levesque et al., 2014b) and men's emotional intelligence are associated with higher DC. Additionally, men's perspective taking predicts women's DC and women's empathic concern can predict men's DC (Levesque et al., 2014a). Couples with higher relationship-focused standards (Wunderer and Schneewind, 2008), a passionate love style (Gagliardi et al., 2015), functional types of couples (validating, volatile, and conflict avoidant) (Bodenmann et al., 2004), and securely attached couples (Gagliardi et al., 2013) rely more on DC. Rational love styles predict more positive DC only in women in in Swiss and German couples (Gagliardi et al., 2015). DC has also been shown to be beneficial for other family members. Zemp et al. (2016) found that DC predicted lower internalizing and externalizing symptoms and higher prosocial behavior in children, with particularly stable effects for externalizing behavior.

### Coping With Medical and Mental Health Conditions

All studies in Western populations have found an association between DC and positive individual indicators in both patients and their partners in couples coping with a medical or mental health condition. DC has been linked to physical well-being in women with breast cancer (Feldman and Broussard, 2006) and less psychological distress and higher quality of life in European couples coping with COPD (Meier et al., 2011; Vaske et al., 2015).

Similarly, studies on relational outcomes have consistently suggested benefits of DC. In Western couples DC has been associated with increased relationship satisfaction in parents raising Autistic children (Gouin et al., 2016) and better partner acceptance and relationship satisfaction in women with breast cancer (Zimmermann et al., 2010). When a partner is diagnosed with PTSD, low discrepancies between partners' DC also predict better relationship satisfaction regardless of the severity level of the PTSD (Witkovsky and Braakmann, 2015). Again, overall DC has been found to have positive effects on other family members beyond the partners. Parents' DC has been linked to better health outcomes in children with type 1 diabetes in German families (Körner et al., 2013). Factors decreasing Western couples' use of DC strategies include traumatic events, depression, anxiety, and COPD (e.g., Gabriel et al., 2016).

## Stress Communication (STM)

Despite the fact that several DC models include stress communication as important aspect of the DC process, it is mostly the STM that has guided the study of this DC dimension. This may be due to the fact that, first, it is explicitly included in the STM conceptualization of DC and second, the DCI, the STM based self-report instrument, specifically includes items to assess this dimension.

### Coping With Non-medical Stressors

Stress communication has consistently been found to benefit couple relationships, as it is associated with increased likelihood of both male and female partners providing support (e.g., Bodenmann et al., 2015) and better relationship satisfaction in Japanese (Yokotani and Kurosawa, 2015), Latino (Falconier et al., 2013b), and Western European and American couples (e.g., Ledermann et al., 2010; Levesque et al., 2014a). It has also been related to constructive communication in European (e.g., Ledermann et al., 2010) and Latino (Falconier et al., 2013b) couples. Additionally, stress communication is associated with positive individual coping in both men and women (e.g., Falconier et al., 2013b). A micro-analytic longitudinal study also showed that the type of stress communication is directly linked with the subsequent coping reaction even in small time frames (Kuhn et al., 2017). It has also been found that unhappy couples seem to rely more on factual stress communication and less on emotional exchanges (Bodenmann and Perrez, 1991).

### Coping With Medical and Mental Health Conditions

Studies with Western couples in which one partner suffers from depression (Bodenmann et al., 2004) or cancer (e.g., Weißflog et al., 2016) have indicated that patients tend to communicate about their stress less frequently than their partners do. It is possible that depressed patients might suffer from a lack of energy, generally employ maladaptive coping strategies (Kovacs and Beck, 1978), and thus experience a decline in their communication competences (Hoffmann et al., 2016), whereas patients with cancer might consciously hold back information that would make their partner worry. Nonetheless, stress communication with medical conditions has been found to have a positive effect individually, improving COPD patients' quality of life (Vaske et al., 2015) and to trigger the provision of support by the healthy partner (e.g., Badr et al., 2010).

## Individual Positive DC: Delegated DC (STM)

Delegated DC, one of the positive ways to help a partner cope with stress, has been included only in STM studies as it is part of its conceptual model and its measurement instrument, the DCI. Compared to other DC dimensions there are fewer studies that specifically focus on delegated DC.

### Coping With Non-medical Stressors

Studies on couples coping with stress in general show that providing delegated DC is positively associated with individual positive coping strategies for both men and women in Latino (Falconier et al., 2013b) and Romanian couples (Rusu et al., 2016). Delegated DC is also linked to constructive conflict resolution and relationship satisfaction for Latino (e.g., Falconier et al., 2013b) and Western European couples (e.g., Vedes et al., 2013), and exclusively to relationship satisfaction for Canadian and American couples (Randall et al., 2015) and Japanese men (Yokotani and Kurosawa, 2015). Nonetheless, when compared with other DC dimensions delegated DC is less strongly linked to marital communication (Ledermann et al., 2010) and relationship satisfaction (for a review see Falconier et al., 2015).

### Coping With Medical and Mental Health Conditions

Delegated coping is often studied in the context of physical or psychological conditions, probably because in the context of chronic illness it is expected that one of the ways in which partners can support the ill partner is by taking over some of their tasks. Logically, in the context of illness, it would be expected for the non-ill partner to provide more delegated DC than the sick partner. For example, COPD or cancer patients report engaging in delegated DC less frequently than their partners do (e.g., Meier et al., 2012). However, this imbalance may not be necessarily beneficial as patients with COPD report a lower quality of life when there is a higher imbalance in partners' delegated DC (Meier et al., 2011). Furthermore, another study on Danish couples coping with breast cancer found that while providing delegated DC to the patient lowers the partner's depressive symptoms, patients tend to report more depressive symptoms when they provide more delegated DC to their partner (Rottmann et al., 2015). These findings suggest that in couples coping with illness imbalance in delegated DC between partners might be beneficial but only to a certain extent.

## Individual Positive DC: Empathic Responding (RFM)

Empathic responding is part of the RFM and is one of the positive ways in which an individual may help a romantic partner cope with stress. Unfortunately, only a few studies have examined this DC dimension. Additionally, studies on empathic responding have measured this construct without discriminating between cognitive/affective and behavioral strategies and therefore, it is not possible to report on the effects of each set of responses.

### Coping With Non-medical Stressors

Only one study examined empathic responding when coping with stress in general. This study (O'Brien et al., 2009) investigated Canadian stepfamilies using a daily dairy methodology and found that both partners perceived lower marital tension on the days following the use of empathic responding. However, husbands' use of empathic responding was associated with increased perception of same-day marital tension while the opposite was true for wives, suggesting gender differences in the use of empathic responding.

### Coping With Medical or Mental Health Conditions

Three studies have examined empathic responding in the context of medical or mental health conditions. The first examined couples coping with the male partner's Alzheimer's disease (Kramer, 1993) and found that partners' empathic responding was related to higher satisfaction in women's caregiving. A second study was a cross-sectional examination of Canadian couples with children with disabilities (Marin et al., 2007), which found that empathic responding is not linked to psychological well-being unless the individual perceives that his or her empathic responding is not reciprocated by the partner, in which case it is associated with psychological distress. The third study (Lee-Baggley et al., 2005) indicated that the individual's conscientiousness, openness, and extraversion contributed to empathic responding in couples coping with child misbehavior while the opposite was true for agreeableness and there was no link to neuroticism.

## Individual Positive DC: Active Engagement (RFM)

Active engagement, which is a positive way to assist a stressed partner, is an RFM concept that was developed in the context of couples coping with an illness. As a result, active engagement has been examined mostly in that context.

### Coping With Non-medical Stressors

Only one study has examined active engagements as a DC strategy for couples to manage stress in general. In that study Kurosawa et al. (2015) found that in Japanese couples with pre-school children active engagement was linked with higher relationship satisfaction.

### Coping With Medical and Mental Health Conditions

Most of the studies on active engagement have been conducted in relation to cancer in the Netherlands (e.g., Kuijer et al., 2000; Hinnen et al., 2009). Other medical conditions studied in relation to active engagement in couples have included Type-II diabetes in American couples (e.g., Schokker et al., 2010), heart problems in Israeli (Vilchinsky et al., 2011), and Dutch couples (Joekes et al., 2007). Across these various medical conditions several studies have found active engagement to have positive effects on the couple's relationship and either no effect or a positive effect on the individual. When partners become actively engaged, both patient and partner report better relationship satisfaction (e.g., Schokker et al., 2010), better individual coping with the illness, lower distress, higher self-efficacy, better health-related quality of life (Coyne and Smith, 1991, 1994; Kuijer et al., 2000; Joekes et al., 2007), and decreased smoking (Vilchinsky et al., 2011). Partners' active engagement has also been found to moderate the negative association between protective buffering and relationship satisfaction (Schokker et al., 2010) in patients with diabetes. Additionally, partners seem to use active engagement more than patients do (Lavery and Clarke, 1999). However, when both patients and partners use active engagement, they report better marital adjustment (Badr, 2004).

Only two studies found active engagement to be unrelated to individual outcomes. Hinnen et al. (2009) reported that partner's active engagement was not associated with cancer patients' distress, regardless of their perceptions of received support or their feelings of mastery. Similarly, Sormanti et al. (1997) found that partner's active engagement was unrelated to quality of life, depression, or health care behavior. Among factors affecting active engagement negativity about the prognosis in cancer patients was found to increase it (Kuijer et al., 2000).

## Individual Positive DC: Supportive DC (STM-DCCM)

Supportive DC is one of the positive ways in which an individual experiencing stress can be helped by a romantic partner. It has been conceptualized within the STM and thus measured with the DCI (Bodenmann, 2008). However, DCCM includes a dimension of partner's supportiveness that is consistent with the STM's definition of supportive DC and has been mostly applied in the study of couples coping with chronic illness.

### Coping With Non-medical Stressors

Studies suggest that individuals who provide emotion- and problem-focused support to a stressed partner are also more likely to use positive individual coping strategies (e.g., Randall et al., 2015) and report increased well-being (Rusu et al., 2015). In terms of couple benefits, supportive DC is linked to relationship satisfaction in Latino (Falconier et al., 2013b), European (e.g., Ledermann et al., 2010), American (Randall et al., 2015), and Canadian couples (Levesque et al., 2014a) and in Japanese husbands (Yokotani and Kurosawa, 2015). Interestingly, for one partner's relationship satisfaction, the subjective *perception* of how much supportive DC the partner provides seems more important than how much the supporting partners themselves indicate providing. A partner could thus provide only little support, yet, the relationship satisfaction is rather linked to what the receiving partner thinks he or she is receiving (Landis et al., 2013). Additionally, in Western couples supportive DC is related to sexuality, romance and passion, constructive conflict resolution and communication, shared meaning (e.g., Ledermann et al., 2010; Vedes et al., 2013), and relationship stability (Bodenmann and Cina, 2005). Men's supportive DC has also been found to buffer the negative effects of the female partner's immigration stress on relationship satisfaction in Latino couples living in the U.S (Falconier et al., 2013a). Regarding the developmental course of supportive DC, in a study on German couples Johnson and Horne (2016) found that supportive DC predicted significantly future commitment and willingness to sacrifice within 5 years, but not the other way around, indicating that supportive DC enhances relationship functioning. However, the same study (Johnson et al., 2016), found a constant decline in supportive DC over time. In young couples, however, male's more rapid decline in supportive DC was associated with a slower decline in women's supportive DC.

In terms of factors that affect providing supportive DC, a spiritual orientation favors the use of supportive DC in Latino couples (Austin and Falconier, 2013) while a traditional gender role orientation in men has the opposite effect (Falconier, 2013). Economic pressure has also been found to reduce couples' use of supportive DC over time (Johnson et al., 2016). Severe depression decreases the use of supportive DC in Swiss couples (Bodenmann et al., 2004).

### Coping With Medical and Mental Health Conditions

Studies have shown both positive and negative effects of supportive DC on the individual. Supportive DC has been linked to less distress in breast cancer patients and their partners (Badr et al., 2010) and individual positive self-verbalization as well as problem-solving in couples with a currently or formerly depressed partner (Bodenmann et al., 2004). In Dutch couples with colorectal cancer, perceived spousal supportive behavior has been a negative predictor of distress over time but only for patients low in perceived personal control; couples with a high sense of personal control reported lower levels of distress 6 months later, regardless of partner support (Dagan et al., 2011). However, one study found that receiving supportive DC could increase depressive symptoms in women with breast cancer (Rottmann et al., 2015).

At a relational level, supportive DC has been associated with relationship satisfaction in Spanish couples with an autistic child (García-López et al., 2016) and in American couples coping with cancer (Checton et al., 2015). A study on American civilian women and their combat veteran partners also found that the negative association between the veteran's post-traumatic stress and their female partner's relationship satisfaction could be buffered the higher women indicated their partner's supportive DC (Lambert et al., 2015).

## Conjoint DC: Collaborative/Common/Communal DC and Mutual Responsiveness (DCCM—STM—CCM—RCCM)

Conjoint forms of DC are responses to stress experienced by both partners and/or to problems that partners see as sharing (“our” problem) even if they originated in one partner (e.g., an illness). Compared to other DC dimensions, positive conjoint strategies, particularly STM's common DC, and DCCM's collaborative coping, has received the most attention in research. There are only a handful of studies that have looked at communal coping or mutual responsiveness in couples.

### Coping With Non-medical Stressors

Studies on Latino (Falconier et al., 2013b), American (e.g., Randall et al., 2015), and Western European (e.g., Bodenmann, 2000) couples show that, similar to supportive DC, partners that engage in common DC also tend to use effective individual coping strategies. Unlike other DC dimensions, common DC has found to be associated with relationship satisfaction not only in Latino, American, and Western European couples but also in Eastern couples such as Japanese (e.g., Yokotani and Kurosawa, 2015) and Chinese (Xu et al., 2016). In European couples common DC is also linked with sexuality, romance, passion, constructive conflict resolution, shared meaning, and commitment (Ledermann et al., 2010; Vedes et al., 2013; Landis et al., 2014), and less verbal aggression and anger (e.g., Bodenmann et al., 2010b). Compared to supportive DC, common DC is a stronger predictor of relationship satisfaction (e.g., Falconier et al., 2013a) and has stronger moderating effects in the association between different love styles and relationship satisfaction in Swiss couples, particularly for the female partner (Vedes et al., 2016). Common DC also helps work through grief (Bergstraesser et al., 2015) and attenuates the negative effects of posttraumatic stress on relationship satisfaction for American female spouses of combat veterans (Lambert et al., 2015) and of immigration stress on relationship satisfaction for Latino couples (Falconier et al., 2013a). Spirituality and a non-traditional role orientation are related to more frequent common DC in Latino couples (Austin and Falconier, 2013; Falconier, 2013).

A study on communal coping (Lin et al., 2016), indirectly measured through the frequency of *we-*talk, found that Taiwanese wives' *we-*talk was linked to husbands' higher work and marital satisfaction husbands' *we*-talk was only related to wives' work satisfaction.

### Coping With Medical and Mental Health Conditions

Common and collaborative DC have been associated with better individual problem solving and decreased negative emotional expression in currently or formerly depressed individuals and their partners (Bodenmann et al., 2004). They have also been linked to lower depression in both partners when coping with breast cancer in Danish couples (Rottmann et al., 2015) and improved physical well-being in American women with breast cancer (Feldman and Broussard, 2006) and men with prostate cancer (Berg et al., 2011). The study on men with prostate cancer (Berg et al., 2008), based on daily diary data, also reported that collaborative DC was linked with more positive and less negative emotions and individual coping effectiveness in both partners. Nonetheless, the same study also reported that for women, collaborative DC exacerbated the negative emotion co-variation between the spouses. The researchers explained that “one of the potential downsides to collaborative coping for women is that one may bear the brunt of the distress that the spouse is experiencing” but that these “short-term costs of collaboration” were perhaps “associated with more long-term gains as the active management nature of collaborative coping may be associated with long-term relational benefits” (p. 513). However, another study (Berg et al., 2011) that also examined American couples coping with breast cancer found that even though common DC was related to better dyadic adjustment for both partners, it was associated with higher distress in patients. In line with positive findings, communal coping, as measured partners' use of *we* language in, has been associated with lower depression in American women with breast cancer (Robbins et al., 2013) and improved alcohol abstinence during treatment and at follow up in American couples (Hallgren and McCrady, 2016). Spouse's *we*-talk predicted positive change in heart failure symptoms and general health over the following 6 months (Rohrbaugh et al., 2008) and smoking abstinence 12 months after quitting in American individuals with heart or lung disease (Rohrbaugh et al., 2012).

At the relational level, common and collaborative DC have consistently been found to have positive effects in couples coping with medical conditions. It has been associated with perceptions of the partner's acceptance of appearance in German women with breast cancer (Zimmermann et al., 2010), sharing more common goals in American couples with prostate cancer (Berg et al., 2008), and increased relationship satisfaction and/or couple's cohesion in Danish couples coping with cancer (Rottmann et al., 2015) and in Australian couples in which women were at an increased risk for breast/ovarian cancer (Watts et al., 2011). *We-*talk as an indicator of communal coping has been associated with relationship adjustment in American couples coping with breast cancer (Robbins et al., 2013). In Kenyan couples communal coping helped HIV-negative couples try to avoid HIV acquisition and helped zero-discordant couples prevent HIV transmission and lived positively with HIV (Rogers et al., 2016). Consistent with these findings, couples coping with breast cancer that reported mutual responsiveness DC, also reported stronger relationships (Kayser et al., 2007).

## Individual Negative DC: Overprotection (RFM)

This negative form of DC to respond to a partner's stress was introduced by the RFM. It has been studied exclusively in the context of serious medical conditions.

### Coping With Medical Conditions

Except for one study that found no effect of spousal overprotectiveness on patient's adaptation to myocardial infarction and a positive association with the couple's closeness (Fiske et al., 1991), studies have reported overprotectiveness to be associated with negative outcomes, particularly individual ones. Partners' overprotection has been associated with less improvement in self-efficacy in Dutch patients with coronary disease (Berkhuysen et al., 1999), less sense of control and more psychological distress in Dutch cancer patients (Kuijer et al., 2000) and CODP patients (Snippe et al., 2012), worse physical condition in cardiac patients (Joekes et al., 2007; Vilchinsky et al., 2011), and reduced dietary adherence and more diabetes distress in American diabetic patients (Johnson et al., 2015). Regarding relational outcomes, Hagedoorn et al. (2000) found that overprotection was associated with lower marital satisfaction only for cancer patients that were experiencing high psychological distress or physical impairment. Bertoni et al. (2015) also found that when partners in Italian couples overprotected cardiac patients, the patients engaged less in their treatment.

## Individual Negative DC: Protective Buffering (RFM)

Similar to overprotection, this form of DC was introduced by the RFM. It has been studied primarily in the context of couples coping with chronic illness.

### Coping With Non-medical Stressors

Only one study has examined the role of protective buffering in couples in a non-medical context. This study examined Japanese couples with pre-school children (Kurosawa et al., 2015) and found no significant associations of protective buffering with either relationship satisfaction or well-being, suggesting the possibility that protective buffering may play a different role when coping with non-medical stressors. However, the same study found that couples with more serious stressors tended to use protective buffering as a coping strategy more often.

### Coping With Medical Conditions

Protective buffering has been studied in American couples with medical conditions such as heart and/or lung problems (e.g., Butler et al., 2014), Type-II diabetes (Johnson et al., 2014), and stem cell transplantation (Langer et al., 2009), in Dutch couples with cancer (e.g., Hagedoorn et al., 2011), heart problems (Joekes et al., 2007; Vilchinsky et al., 2011), CODP (Snippe et al., 2012), and diabetes (Schokker et al., 2010), and Australian couples with cancer (Lavery and Clarke, 1999). In the context of medical stressors both the patient and his/her partner may try to help each other cope through protective buffering (e.g., Langer et al., 2009). However, findings have been inconsistent regarding who relies more on this coping strategy. Some studies have found that caregivers tend to use more protective buffering than their ill partners (e.g., Langer et al., 2009), whereas other studies reported the opposite (e.g., Manne et al., 1999).

Regardless of which partner provides protective buffering and despite seemingly positive intentions, protective buffering has negative effects on individual and relational well-being for both providers and recipients in Western couples dealing with a medical condition. Receiving protective buffering has been associated with (a) lower physical exercise and glycemic control in diabetes patients (Johnson et al., 2014), (b) poorer mental health in recipients of stem cell transplants (Langer et al., 2009), (c) depression in men with heart disease (Hagedoorn et al., 2011; Vilchinsky et al., 2011), (d) distress in cancer patients (Manne et al., 2012), and (e) lower relationship satisfaction (e.g., Langer et al., 2009), particularly when there was low partner support for cancer patients (Hagedoorn et al., 2011). Even women undergoing genetic tests for cancer reported greater distress 6 months after receiving protective buffering (Manne et al., 2004). Furthermore, in the presence of overprotection, which partners also tend to use when they engage in protective buffering (e.g., Kuijer et al., 2000) only protective buffering is significantly associated with distress in patients with COPD (Snippe et al., 2012). Similarly, providers of protective buffering experience lower relationship satisfaction (Hinnen et al., 2008; Schokker et al., 2010), and greater distress regardless of whether they are patients or caregivers (Suls et al., 1997; Manne et al., 2007). A study of couples' coping with lung cancer indicated that patients that engaged in protective buffering reported higher pain severity and fatigue and poorer mental health (Lyons et al., 2016).

There are only a few exceptions to this pattern of results: Badr (2004) found that when American couples are more congruent in using active engagement but more complementary in the use of avoidance coping and protective buffering, they tend to report greater marital quality. Another study found that caregivers reported higher relationship satisfaction when they provided protective buffering (Langer et al., 2009). Regarding factors affecting protective buffering, one study of couples coping with cancer found that lower life expectancy increases the use of protective buffering (Manne et al., 1999).

## Individual Negative DC: Hostile/Ambivalent DC (STM)

In our integration of DC models, STM's form of negative DC has been addressed as hostile/ambivalent. In this way other negative DC dimensions (e.g., overprotection) can also be considered independently of the one introduced by the STM.

### Coping With Non-medical Stressors

Hostile/ambivalent DC has been linked with negative individual and relational functioning. At the individual level this DC dimension is associated with higher verbal aggression, anger, insomnia, depression, men's physical symptoms, and women's social dysfunction in Swiss couples (Bodenmann et al., 2010a, 2011) and catastrophizing in Romanian couples (Rusu et al., 2016). No association has been found with individual positive forms of coping (e.g., Randall et al., 2015). At the relational level hostile/ambivalent DC is linked to lower marital quality in both partners in European couples (e.g., Vedes et al., 2013), American couples (Randall et al., 2015), Latino couples in the U.S (Falconier et al., 2013b), Canadian couples (Levesque et al., 2014a), and Japanese women (Yokotani and Kurosawa, 2015). Hostile/ambivalent DC has also been negatively associated with sexuality, romance, passion, and constructive conflict resolution in Portuguese couples (Vedes et al., 2013) and has been found to be a stronger predictor of lower relationship satisfaction than individual coping in American couples (Papp and Witt, 2010). In our integration of DC models, the STM's form of negative DC has been addressed as hostile/ambivalent so that other negative DC dimensions (e.g., overprotection) can also be considered independently of the one introduced by the STM.

In terms of factors affecting the use of use hostile/ambivalent DC, a study on Italian couples found that parents' use of this form of negative DC increases the likelihood that their children will use it similarly in their romantic relationships (Donato et al., 2012). Additionally, rational love as compared to passionate love is associated with men's hostile/ambivalent DC (Gagliardi et al., 2015) and traumatic events exacerbate the use of hostile/ambivalent DC in Swiss couples (Kramer et al., 2005). By contrast, secure attachment is linked to less frequent use of hostile/ambivalent DC (Gagliardi et al., 2013).

### Coping With Medical or Mental Health Conditions

In general, hostile and ambivalent DC has been linked to negative outcomes for the individual and the relationship in couples coping with physical- and mental health-related stressors across different Western cultures. In German couples coping with COPD, use of hostile/ambivalent DC is related to lower quality of life (Vaske et al., 2015). German couples coping with a partner's hematologic malignancy reported higher unmet supportive care needs when hostile/ambivalent DC was higher (Weißflog et al., 2016). When coping with breast cancer, hostile/ambivalent DC was associated with partners' poorer emotional well-being and patients' poorer physical well-being in patients in American couples (Feldman and Broussard, 2006), and with depressive symptoms and lower relationship quality in Danish couples (Rottmann et al., 2015).

Various factors predict a more frequent use of hostile/ambivalent DC in couples. Swiss couples coping with COPD use hostile/ambivalent DC more frequently than healthy couples do (Meier et al., 2012), and an imbalance in delegated DC increases the likelihood of relying more on this negative DC form (Meier et al., 2011). Swiss couples in which one partner is depressed/was formerly depressed (Bodenmann et al., 2010a) or who have children with externalizing behaviors (Gabriel et al., 2008) also use hostile/ambivalent DC more often than couples without a depressed member or a child with externalizing behaviors. Similarly, caregiving burden predicted more frequent hostile/ambivalent DC in Canadian couples with autistic children (Gouin et al., 2016).

The only exception to this link between hostile/ambivalent DC and negative individual indicators is an Italian study conducted by Bertoni et al. (2015). In this study, when couples used hostile/ambivalent DC to cope with cardiac problems, the cardiac patient's partner was more engaged in the problem.

## Demographic, Cultural, and Contextual Factors: Gender, Age, and Culture

Several studies on Western populations have found that both partners report women as engaging more frequently in positive forms of coping such as providing delegated DC, supportive DC, and common and collaborative DC (e.g., Bodenmann et al., 2010a; Falconier et al., 2013b; Zeidner et al., 2013). Even though results have been inconsistent regarding which partner has a more positive evaluation of their overall couple's coping strategies (e.g., Vedes et al., 2013), DC also plays a more important role for women's relationship satisfaction than for men's in many Western cultures (e.g., Gmelch and Bodenmann, 2007; Papp and Witt, 2010). Additionally, several studies have found that women communicate their stress more often than men in Western couples (e.g., Donato et al., 2009). Some studies have also reported men to be more likely than women to provide negative DC forms such as protective buffering (Manne et al., 1999) and hostile/ambivalent DC (e.g., Yokotani and Kurosawa, 2015). This is consistent with the finding that lesbian couples reported receiving better DC and experiencing less conflict when compared to heterosexual couples (Meuwly et al., 2013). Nonetheless, some studies have failed to find any gender differences in some DC positive forms such as delegated DC (e.g., Rusu et al., 2016) or supportive DC (García-López et al., 2016).

Studies have reported inconsistent findings regarding the effect of age or length of relationship on overall DC or any of its dimensions, regardless of whether the study focused on coping with general stress or on medical or mental health conditions. Some studies found no effect of age or relationship length on overall DC (e.g., Ruffieux et al., 2014; Reed et al., 2016), stress communication (e.g., Levesque et al., 2014a), delegated DC (e.g., Levesque et al., 2014a), active engagement (e.g., Joekes et al., 2007), protective buffering (Langer et al., 2009). By contrast, other studies have reported that younger couples engage more frequently in positive forms of DC than older couples including overall DC (e.g., Meyer et al., 2005), active engagement with a sick partner (e.g., Schokker et al., 2010), and supportive DC (e.g., Levesque et al., 2014a) and less frequently in negative DC forms such as overprotection (e.g., Joekes et al., 2007) and that older couples rely more frequently on factual stress communication than younger couples do (Bodenmann and Widmer, 2000). Regarding length of relationship, several studies have found a positive relation between length of relationship and DC negative forms such as protective buffering (Schokker et al., 2010) and hostile/ambivalent DC (e.g., Yokotani and Kurosawa, 2015), but one study found also a positive association for overall DC and common or collaborative DC (e.g., Feldman and Broussard, 2006).

When analyzing results from all DC studies, given that the majority of those studies have been conducted with European couples, it is not possible to reliably identify a pattern of results that could be indicative of cultural differences between Western and Eastern populations or even between Western European and non-European couples (e.g., Latin American couples). Evidence from two studies suggests that stress communication might be a DC dimension in which Western and Eastern couples manage differently. One study on Chinese couples (Xu et al., 2016) reported that men communicated stress more frequently than women did, which was in sharp contrast with the great number of Western studies that have consistently found the opposite gender pattern. A second study found that intercultural Thai-Swiss couples communicated about their stress less frequently than mono-cultural Swiss couples (Gagliardi et al., 2010).

Only a handful of studies have actually looked at the role culture in the couples' stress and coping process by examining different cultural groups in the same study. As described earlier, Kayser et al. (2014) interviewed American, Chinese, and Indian couples coping with breast cancer and concluded that compared to American couples, Asian couples viewed the illness as beyond their control and they were therefore more inclined to accept it rather than desperately trying to do something to change it. Additionally, Asian couples had more gender differentiated roles and involved their families in their coping efforts (open boundaries) more often. Asian couples also coped in ways that showed more interdependence. Another study comparing three different cultural groups found that Chinese couples reported significantly less delegated DC than Swiss and American couples (Xu et al., 2016). This set of findings suggests that Asian couples cope in ways congruent with their collectivistic orientation whereas American couples cope in ways consistent with their individualistic orientation.

A large recent, cross-sectional study across 35 different countries (Hilpert et al., 2016) found that supportive and common DC considered together predicted relationship satisfaction across all nations. It also found that couples in African countries used supportive and common DC more frequently than couples in Asian countries such as Hong Kong and South Korea. However, the study yielded two interesting findings. First, results did not support differences between Eastern and Western cultures in the association between DC and relationship satisfaction. For example, Nigeria, India, Ghana, Iran, Portugal, and Kenya were among the countries with the smallest effect of DC on relationship satisfaction and Bulgaria, Romania, Hong Kong, Slovakia, and Canada where among the countries with larger effects, indicating significant variability within a region whose countries were expected to be culturally related. Then, the size of the effect of DC on relationship satisfaction was independent from the frequency with which they used supportive and common DC. For example, couples from Bulgaria, Canada, and Greece reported using DC frequently and that coping behavior had a large impact on relationship satisfaction, whereas couples in Ghana, Kenya, and Nigeria were also high in DC behaviors but their coping had a small effect on the relationship.

## Discussion

### The DC Conceptual Integration

The conceptual review and integration of the various DC models presented in this paper suggests that the various theoretical frameworks that have been developed can be brought together to present a more comprehensive picture of the DC process. Even though DC models differ in its origins with most of them developed to understand the couple's process to cope with a medical condition and one, the STM, to explain how couples cope with everyday stress, most models have been applied to examine DC with medical and non-medical stressors, providing support for the integration of all DC models into a larger framework that can explain all DC processes. The integration is also possible because there are no contradictions among the various DC models. To begin with, they all share a systemic perspective in which each partner's experiences of stress and coping with external stressors are interrelated. In addition, all DC models support the same chore principles about the DC process: (a) stress appraisals (“my” or “your” vs. “our” problem) shape DC responses; (b) partners communicate about their stress; (c) partners engage in DC individual strategies that can help the other partner cope with stress or in conjoint strategies to handle stress together; (d) DC may be positive or negative; and (e) relationship and contextual factors may affect stress appraisals and DC. DC models only differ on the attention given to each of those chore principles and the different types of individual and conjoint coping strategies.

Nonetheless, the integrative DC model advanced in this paper should continue to be expanded and refined conceptually. It is possible that new dimensions are identified beyond the ones included at present. However, new theoretical developments could consider the present integration to avoid construct overlaps and conceptual fragmentation in the field. The present integrative model should keep being expanded to also incorporate the work in related fields such as emotional co-regulation and spousal support.

### The Review of the DC Empirical Literature

Following the conceptual integration of DC models, the goal of this narrative review was to present the findings from all studies on DC and each of its specific dimensions in order to provide a complete picture of the accumulated empirical knowledge and suggest areas in need of research.

This empirical review, which includes mostly studies conducted on American and European populations, suggests that taken together or separately, most dimensions of positive individual (helping partner cope with stress) and conjoint forms (partners coping together with stress) of DC are associated with better individual and relational functioning when coping with either medical or mental health conditions or other types of stressors, while the opposite is true for negative individual DC strategies In other words, when couples report using DC, empathic responding, active engagement, supportive DC, delegated DC, stress communication, or common or collaborative DC they also tend to report higher use of effective individual coping strategies, higher life satisfaction, lower psychological distress, and depression when coping with stress in general and better illness management, health related quality of life, improved physical and emotional wellbeing when coping with a mental health or medical condition. At a relational level, these couples tend to report more constructive communication, sexual and relationship satisfaction, commitment, and stability over time when coping with stress in general as well as with mental health and medical stressors. When couples use positive forms of coping they can also buffer the negative effects of stress on their individual levels of aggression and on their relationship satisfaction. In contrast to positive forms of DC, all three forms of negative DC have been related to negative individual and relational functioning. In the context of illnesses, regardless of which partner is the provider or recipient, overprotection, protective buffering, and hostile/ambivalent DC are linked to lower self-efficacy, sense of control, physical and emotional well-being, and relationship satisfaction. Similarly, when couples use hostile/ambivalent DC to cope with stress in general, they report more destructive communication and conflict resolution, and relationship dissatisfaction.

Despite this overall picture that suggests that there are individual and relationship benefits to using positive DC strategies but risks to relying on negative forms of DC, further research is necessary to better understand the DC process. Findings included in this review suggest that some DC dimensions may be more critical than others in harming or protecting individual and relational well-being. For example, common and collaborative DC tend to be more beneficial than other positive DC dimensions for both medical and non-medical stressors while delegated DC seems to be the least beneficial. Furthermore, it is possible that some DC forms might not even be beneficial across all stressful situations. For example, delegated DC does not seem to be positive for partners with medical conditions and imbalanced delegated DC between the partners is negative for patients. Similarly, a lack of reciprocation in empathic responding or supportive DC is linked to psychological distress. This set of findings indicate that not all DC strategies are equally beneficial or negative across different contexts. However, further research is necessary to examine differential effects of all DC strategies and variations across different stressors. As a illustration, it might be that controlling DC or overprotection turn out to be less negative when coping with medical conditions than when coping with other type of stressors or that active engagement has positive effects only when coping with medical conditions but not when a partner is coping with a stressful situation that he or she might want to avoid sharing with a partner or feels responsible for (e.g., conflict with a family member, job-related problem, etc.). In order to answer some these questions, more attention should be given to examine forms of DC that have been understudied such as conjoint negative DC, controlling DC, or empathic responding. In addition, DC forms should be studied across different stressful contexts. For example, most studies on medical stressors are on couples coping with cancer and therefore, further research could focus on the role of DC and each of its dimensions in couples coping with other medical conditions. Similarly, studies should also examine DC in couples coping with other non-medical stressors such as economic problems, immigration related issues, raising children with disabilities, emotional and/or behavior difficulties, caring for elderly family members, etc. Researchers should specially focus on examining DC dimensions that have only been studied in relation to only one type of stressor. As an illustration, overprotection has only be examined in the context of medical illness.

Additionally, improvement in design and measurement instruments could further advance the field and provide more reliable findings. More than 100 of the 139 articles included in the present review were reports of cross-sectional studies, precluding conclusions on causal direction. Longitudinal studies did indicate that it is DC that predicts many of the individual and relational outcomes but clearly more longitudinal studies are needed to provide further support. Also, and most importantly, except for 18 studies all studies relied on self-report questionnaires. Unfortunately, this is an important methodological constraint to the study of DC. First of all, some self-report measures (e.g., DCI) assess dyadic coping with stress in general and not in relation to a specific stressor, assuming that couples rely on DC to the same extent and in the same way across different contexts of stress. These measures are even more problematic when used in studies on couples coping with a particular stressor as it is not possible to know whether the responses on coping apply to the way in which the couple is managing that stressor. Then, even if the self-report measure is specific to the stressful situation, asking partners about their overall impression on DC assumes that there is stability over time and consistency in how couples cope with various types of stressors. However, the developmental perspective introduced by the DCCM in studying couples coping with illness suggests that couples' coping can change over time. This limitation might be overcome by the use of daily diaries, which would allow to see changes in DC over time and/or by stressor.

Last, and also well-known are the biases introduced by self-reports. The few studies that have been conducted in the DC field relying on observational and physiological data have allowed not only to obtain less biased data on partners' use of DC and its effects on each other but also to micro-analyze the DC process following moment-to-moment interactions instead of assessing DC at a macro level. This type of micro-analysis has helped linked, for example, differences in stress communication with particular partner's responses. For example, Kuhn et al. (2017) found that problem-oriented stress expression was strongly linked to problem-oriented dyadic coping in a time sequence of 10 s within a conversation, while emotion-oriented stress expression was associated with emotion-oriented dyadic coping reactions. Continuing to employ observational and physiological measurement in research may refine our understanding of DC, particularly regarding stress communication, partners‘DC responses, and the effects of DC responses on each partner and their further stress communication and use of DC.

In terms of factors affecting the use of DC strategies, the current review indicates that age, individual psychological and relationship variables, family context, and gender may play some role. Being young, empathic, emotionally intelligent, securely attached, or spiritually oriented and having relationship-focused standards all contribute to using positive forms of DC, whereas experiencing trauma, depression, and/or anxiety, and being older do not. By contrast, depression and having children with psychosocial challenges may contribute to more use of negative DC and imbalance in delegated DC. Western women tend to engage more in positive forms of DC whereas men use more negative forms. Furthermore, DC is more significant for relationship functioning for Western, African, and Asian women and for their male partners. These findings reinforce the need to further study the role of demographic and individual and relationship factors in the use and effects of DC in general and across different stress contexts. This research could explain why men are less likely than women to use DC strategies despite the fact that they also benefit from them or whether age and gender affect DC similarly across different stressful circumstances.

Nonetheless, the review clearly indicates that one of the areas in which the DC field seriously needs to further advance is in the examination of cross-cultural variation. Despite the fact that the cultural context has been included in various DC models (the STM, the DCCM, and the RCCM) as a factor that shapes the stress and coping process and the fact that one study indicated that DC is beneficial for the couple's relationship at least in 35 different countries (Hilpert et al., 2016), most of the research included in the present review have been conducted in Western Europe. The few studies that have examined cultural factors suggest that Asian couples cope in more collectivistic ways, whereas Western couples seem to cope in more individualistic ways. It also appears that some cultures (e.g., African) use DC more frequently than others (e.g., Asian) and that they may also differ on the extent to which their DC behaviors contribute to their relationship satisfaction regardless of DC use. Those findings call for more studies to be conducted in non-Western European populations so that differences in stress appraisal, stress communication, and use and effects of DC strategies can be understood in different cultures.

The other area of inquiry that has not received much attention in the field is DC in the context of same-sex couples. Only one study on same-sex couples was included in the present review (Meuwly et al., 2013) and it indicates that the DC quality might be better in than in heterosexual couples. Further research should be conducted to fully understand the DC process in gay and lesbian couples.

In terms of the interplay of individual coping styles and their effects on the relationship, the review of studies on similarities between partners' styles of coping with their own stress did not show consistent results, suggesting that the fit or congruence between partners' styles may matter more in terms of the impact on the individual and the relationship. However, studies still need to provide evidence for this possibility

The knowledge gained in the field about the individual and relational benefits of positive DC and the harmful effects of negative DC as well as the factors that promote positive DC have had important clinical and programmatic implications. Several interventions have been developed to help couples cope with stress together based on the concepts and empirical findings reported in the present review. A report of all interventions is beyond the scope of this review but The Couples Coping Enhancement Training (CCET; Bodenmann and Shantinath, 2004) to prevent relationship distress by teaching couples cope with stress, the Coping-Oriented Couple Therapy (Bodenmann et al., 2008) that provides a clinical intervention focused on DC, or the TOGETHER program (Falconier, 2015) to assist couples cope with financial stress are just some illustrations of the programmatic and clinical applications of DC models. Further advances in the DC field may provide critical information to design interventions and programs that can reach ethnically diverse couples, different age, religious, and socio-economic groups, same-sex couples, and both men and women.

## Limitations

Despite the contributions of the present narrative review, there are also some limitations. The review only included journal articles published in English and German, which may have left out studies published through other outlets and in other languages. However, compared to other reviews, this review has included the largest number of studies and DC models, offering a more complete picture of the DC field.

Even though we provide supplementary material describing the sample type and size, measures, design, and the main findings of each of the studies reviewed, due to publication length limitations it is not possible to describe or integrate all studies with enough level of detail. Last, this review did not entail a critical analysis of the study designs or a statistical analysis (e.g., meta-analysis) due to the heterogeneity of variables, low number of studies for some of the variables, and need to include qualitative studies. The inherent limitations of studies using cross-sectional and self-report data, which comprise the vast majority of studies in this review, are well-known and have been addressed in our discussion as well.

## Conclusion

Various DC models have been introduced in the last two decades. Even though each DC model has made unique contributions to the understanding of the DC process, conceptual overlap also exists across models. Given that several chore principles are shared across those models, a conceptual integration was possible. The integrative model proposed in this paper includes all the DC dimensions identified by such DC models as well as factors that affect the coping process. The review of all studies applying any of the DC models suggest that in Western couples, positive forms of coping, whether individual or conjoint and taken together or separately, are beneficial for each partner's individual and relational well-being when they cope with stress in general and/or mental health or medical stressors. Few studies in non-Western populations suggest similar benefits. Research on DC can be expanded to include other populations and stressors and use better designs. The accumulated knowledge in the field already has already offered enough guidance for prevention programs and clinical interventions. However, such knowledge should be taken with caution given the design and measurement limitations of the studies as well as the characteristics of the samples.

## Author Contributions

MF came up with idea and design of the study. She conducted a lot of the coding in English and was responsible for the majority of the conceptual review and integration. RK contributed with all the coding in German and writing about the methodology for the review of empirical studies and writing about STM and the empirical findings for STM and DCCM. MF also wrote about the empirical findings of CM, RFM, CMM, and RCCM and reworded STM findings. Both MF and RK worked on creating the table of the summary and findings.

### Conflict of Interest Statement

The authors declare that the research was conducted in the absence of any commercial or financial relationships that could be construed as a potential conflict of interest.
