# Supplementary material for: Dyadic Coping in Couples: A Conceptual Integration and a Review of the Empirical Literature
Source: Front Psychol. 2019 Mar 26;10:571. doi: 10.3389/fpsyg.2019.00571 (PMC6443825; doi:10.3389/fpsyg.2019.00571)
Supplement: Supplementary file 1 [file Table_1.docx]

Table 1 (Online supplemental material). *Summary of findings of study* (*by Dyadic Coping Model)*

|  | | | | | | | | |  |
| --- | --- | --- | --- | --- | --- | --- | --- | --- | --- |
| **Model** | **Study** | **Sample** | **Measures** | | | | **Design** | **Main Findings** |  |
| CM | Barbarin, Hughes, & Chesler (1985) | 32 US couples with children w/ cancer | Coping Inventory created by authors | | | | Cross-sectional | Dissimilarity in problem-focused predicted better marital quality and support. Similarity use of emotion-focused coping better predictor of partners' optimism than complementarity. (symmetrical coping) than if just one does it (complementarity). |  |
| CM | Barnoy et al. (2006) | 98 Israelian married couples w/ one partner w/ cancer | Miller Behavioral Style Scale (Miller, 1987) | | | | Cross-sectional | If both partners are “monitors”, better adjustment for female cancer patient. If both partners are “blunters”, better adjustment for male patient but more psychological distress for caregiver was reported. |  |
| CM | Ben-Zur, Gilbar, & Lev (2001) | 73 Israelian couples w/ women w/ breast cancer | 30-item Hebrew version of Inventory of Coping Strategies (COPE; Carver, Scheier, & Weintraub, 1989) | | | | Cross-sectional | When both spouses use emotion-focused coping, more psychological distress and poor functioning in women. Complementarity in emotion-focused coping (denial, venting) but not on problem-focused predicts greater women's greater distress. Wives' avoidance and husband's problem solving predicted wives' depression. |  |
| CM | Cronkite & Moos (1984) | 267 US married couples | Avoidance and Approach Coping Inventory created by authors | | | | Longitudinal, but only wave 2 data analyzed and as cross-sectional | When wives use avoidance coping and men’s approach coping, wives are more depressive and men report more physical symptoms. When both partners used avoidant coping, men are more depressive. |  |
| CM | Fagundes, Berg, & Wiebe (2012) | 59 US married couples with man w/ prostate cancer | Avoidance scale of the Impact of Events Scale (Horowitz et al., 1979) | | | | Longitudinal (daily diary) | Congruence (similarity) in avoidance was not detrimental for patients’ or wives’ negative affect. Each partner's had positive both actor and partner effects on each partner's negative affect. |  |
| CM | Giunta & Compas (1993) | 153 US couples | WCCL | | | | Cross-sectional | Husbands’ and wives’ coping are associated with each other. Wife’s escape-avoidance predicts wife’s and husband’s psychological symptoms. However, the ability of dyadic coping (similarity or not) to predict symptoms was lacking. |  |
| CM | Hoekstra-Weebers, Jaspers, Kamps, & Klip (1998) | 67 Dutch couples w/child/cancer | The Utrecht Coping List (Sanderman, 1988) | | | | Longitudinal | Similarity in coping styles in parents was found Discrepancies in coping styles positively related to distress in fathers at diagnosis. However, 12 months later, the more discrepant the couples were in their coping preferences the more distress the mothers indicated. |  |
| CM | Holahan, Moos, Moerkbak, Cronkite, Holahan, & Kenney (2007) | 184 US married couples | Coping scale (Moos & Schaefer, 1993) | | | | Longitudinal | Similarity in partners’ individual coping across 10 years and coping similarity strengthened subsequent spousal similarity in depressive symptoms. |  |
| CM | Kraemer, Stanton, Meyerowitz, Rowland, & Ganz (2011) | 139 US couples with women w/ non-metastatic breast cancer | Inventory of coping strategies (COPE; Carver et al., 1989) and the Emotional Approach Coping scales (Stanton, Kirk, Cameron, & Danoff-Burg, 2000) | | | | Longitudinal | Similar use of emotional and problem focused coping predicted women's better adaptation to breast cancer 10 months later. Dissimilarity (women's low emotional coping and men's high emotional coping) predicted higher relationship satisfaction in women later. |  |
| CM | Pakenham (1998) | 45 Australian couples w/ one partners w/ MSI | WCCL | | | | Longitudinal | Dissimilarity in problem-focused coping predicts lower collective depression and better individual adjustment in both patient partner and caretaker partner. Dissimilarity in emotion-focused predicts collective depression negatively and both partners' individual adjustment positively. No association of adaptation with coping similarity. |  |
| RFM | Badr (2004) | 90 US healthy couples and 92 US couples w/ a partner w/ a chronic illness | | Brief COPE (Carver, 1997), RFCS | | Cross-sectional | | Couples who are more congruent in their use of active engagement and more complementary in their use of protective buffering and avoidance coping report greater marital adjustment. | |
| RFM | Berkhuysen, Nieuwland, Buunk, Sanderman, & Rispens (1999) | 114 Dutch individuals w/ coronary disease | | Overprotectiveness (self-developed), 6 items | | Longitudinal, experimental | | Perceived partner's overprotection was associated with lower self-efficacy beliefs to control symptoms. | |
| RFM | Butler, Hollenstein, Shoham, & Rohrbaugh (2014) | 26 US couples w/ one or both partners w/ heart or lung problem aggravated by smoking or at least two other risk factors for coronary artery disease | | Participants rated their regulatory intentions (from protection to engagement) in videotaped interactions with partners based on RFCS | | Intensive longitudinal (state-space grids) | | Use of protective or engagement coping responses is preceded by negative emotional states. Double-smoking couples use more engagement for emotion regulation. Single-smoking couples use more protection to regulate emotions but it interferes with smoking cessation. | |
| RFM | (1) Coyne & Smith (1991); (2) Coyne & Smith (1994) | 56 US married men after an in-patient stay for uncomplicated myocardial infarction | | RFCS | | Cross-sectional | | (1) Active engagement of patients' spouses was associated with decreased distress, while protective buffering was associated with increased distress; (2) Wife’s protective buffering was negatively and active engagement positively associated with patient self-efficacy. | |
| RFM | Fiske, Coyne, & Smith (1991) | 56 US couples w/men w/ a recent uncomplicated myocardial infarction | | Overprotectiveness: The Michigan Family Heart Questionnaire (Fiske et al., 1991) | | Cross-sectional | | Overprotectiveness did not have a linear or curvilinear relationship to patient adaptational outcomes, and it did not interact with a hostile-critical attitude. Over­protectiveness was related to an increase in the couple’s closeness following the myocardial infarction. | |
| RFM | Hagedoorn, Dagan, Puterman, Hoff, Jeroen Meijerink, De Longis, & Sanderman (2011) | 88 Dutch couples in w/one partner w/colorectal cancer | | WGS | | Longitudinal | | When spousal support is low relationship satisfaction is positively associated with active engagement and negatively with protective buffering. | |
| RFM | Hagedoorn, Kuijer, Buunk, DeJong, Wobbes, & Sanderman (2000) | 68 Dutch couples w/ a partner w/ cancer | | WGS | | Cross-sectional | | Positive association between active engagement and the patient's marital satisfaction was stronger for patients with a poorer psychological and physical condition than for those with a better condition. Protective buffering and overprotection negatively associated with marital satisfaction only for patients with high levels of psychological distress or physical limitations. | |
| RFM | Hinnen, Hagedoorn, Ranchor, & Sanderman (2008) | 72 Dutch women w/ cancer and 62 comparison controls and their partners | | Questionnaire on active engagement and protective buffering (Hagedoorn et al., 2000; Kuijer et al., 2000) | | Longitudinal | | Partners' protective buffering related to less relationship satisfaction, especially in more assertive women with cancer but active engagement associated with more relationship satisfaction regardless of assertiveness level. | |
| RFM | Hinnen, Hagedoorn, Sanderman, & Ranchor (2007) | 92 Dutch women w/ breast cancer | | Questionnaire on active engagement and protective buffering (Kuijer et al., 2000) | | Longitudinal | | Small but significant decreases in protective buffering and active engagement over time. Distress and neuroticism predicated protective buffering. | |
| RFM | Hinnen, Ranchor, Baas, Sanderman, & Hagedoorn (2009) | 82 Dutch couples w/ women with breast cancer | | WGS | | Longitudinal | | Active engagement was unrelated to distress but protective buffering reported by partners but unnoticed by patients related to higher women's distress over time. | |
| RFM | Joekes, Maes, & Warrens (2007) | 73 Dutch couples w/ partner in cardiac rehab | | WGS | | Longitudinal | | Partner's active engagement positively predicts later patient's health related quality of life but not physical functioning. Partner's overprotection predicts later worsening of patient's physical functioning. | |
| RFM | (1) Johnson, Anderson, Walker, Wilcox, Lewis, & Robbins (2014) (2) Johnson, Anderson, Walker, Wilcox, Lewis, & Robbins (2015) | 117 US married couples w/ one partner w/ Type 2 diabetes | | WGS | | Cross-sectional | | (1) When diabetes is not perceived as having major consequences, protective buffering is associated with less exercise and worse glycemic control. When partners perceive it as having major consequences, protective buffering is unrelated to frequency of exercise.  (2) Overprotection was associated with reduced dietary adherence indirectly via increased diabetes distress only at low levels of active engagement. | |
| RFM | Kramer (1993) | 72 US couples w/ husbands w/ Alzheimer's disease | | WCCL, relationship-focused coping (O’Brien & DeLongis, 1991) | | Cross-sectional | | Caregivers' relationship-focused strategies associated with more satisfaction in caregiving. | |
| RFM | Kuijer, Ybema, Buunk, Majella de Jong, Thijs-Boer, & Sanderman (2000) | 106 Dutch couples w/ one partner w/ cancer of the larynx or vocal cord and Kahler's disease (bone marrow cancer) | | WGS | | Cross-sectional | | Partners' perception of patient's better coping associated with active engagement and less overprotection. Partner's relationship satisfaction associated with active engagement. Partners' overprotection associated with patients' distress and less control. Partners' protective buffering associated with low high self-efficacy but active engagement with high self-efficacy. | |
| RFM | Kurosawa, Kato, & Kamiya (2015) | 101 Japanese couples w/ at least one pre-school child | | Japanese version of relationship-focused coping (Kurosawa & Kato, 2013; based on Kuijer et al., 2000; Langer et al., 2009) | | Cross-sectional | | When both partners use active engagement couples are more are more satisfied with their relationship. Couples with serious stress use more avoidant and protective buffering coping. Protective buffering no effect on relationship satisfaction and well-being. | |
| RFM | Langer, Brown, & Syrjala (2009) | 80 US couples w/ a partner being a hematopoietic stem cell transplantation recipients | | Protective buffering (Suls et al., 1997; Trost, 2005) | | Longitudinal (before & after transplantation) | | Caregivers higher on protective buffering compared to patients. Patients own and partner buffering was associated with lower relationship satisfaction and poorer mental health. Caregivers that provided protective buffering reported higher relationship satisfaction. | |
| RFM | Langer, Rudd, & Syrjala (2007) | 42 US spousal caregivers of cancer survivors who were 1 year post-transplant | | Oral emotional expression exercises | | Cross-sectional | | Partner’s protective buffering as measured with facial expressions was unrelated to health and relationship outcomes, but lexical (verbal) protective buffering was negatively related to marital satisfaction | |
| RFM | Lavery & Clarke (1999) | 12 Australian couples w/a partner w/prostate cancer | | Coping strategies adopted as individuals and as a couple, communication of emotional needs to spouse (Interviews) | | Cross-sectional, Qualitative | | Spouses exhibit more active engagement, whereas patients employed more protective buffering. | |
| RFM | Lee-Baggley, Preece, & DeLongis (2005) | 71 Canadian couples w/ a w/ a child from a previous union had been residing at home | | Relationship-focused questionnaire (empathy, support provision, compromise, interpersonal withdrawal) | | Longitudinal (daily diaries) | | Relationship-focused coping positively related to conscientiousness, openness, and extraversion (only for child misbehavior as stressor), negatively related to agreeableness and unrelated to neuroticism. | |
| RFM | Manne, Audrain, Schwartz, Main, Finch, & Lerman (2004) | 153 US women and 118 US partners of women undergoing cancer gene testing | | Protective buffering (Manne et al., 1999) adapted from the RFCS | | Longitudinal | | Less support and protective buffering associated with greater distress 6 months postdisclosure among test participants. | |
| RFM | Manne, Badr, & Kashy (2012) | 92 US couples w/ a partner w/ head and neck or lung cancer | | RFCS | | Cross-sectional | | Protective buffering positively associated with distress and general intimacy partially mediated this association. | |
| RFM | Manne, Dougherty, Veach, & Kless (1999) | 121 US cancer patients | | Protective buffering adapted from the RFCS | | Longitudinal | | Male patients and patients with lower life expectancy engaged in more protective buffering. Protective buffering for women, either as patients or wives, predicted more distress and avoidant ideation. Protective buffering did not reduce partner's distress. | |
| RFM | Manne, Norton, Ostroff, Winkel, Fox, & Grana (2007) | 235 US couples w/ women w/ early stage breast cancer | | Protective buffering (Manne et al., 1999), adapted from the RFCS | | Longitudinal | | Protective buffering predicted greater distress over time for the person engaging in protective buffering (patient or partner). These effects are stronger among couples in more satisfying relationships. | |
| RFM | Marin, Holtzman, DeLongis, & Robinson (2007) | 29 Canadian couples w/ children with disabilities | | Empathic responding (O'Brien & DeLongis, 1996) | | Cross-sectional | | If a parent engages in relationship-focused coping and his or her spouse does not reciprocate there is more psychological distress. | |
| RFM | O’Brien, DeLongis, Pomaski, Puterman, & Zwicker (2009) | 82 Canadian couples w/ a child from a previous union had been residing at home | | Empathic responding (O'Brien & DeLongis, 1996) | | Longitudinal, Qualitative (diaries, interviews, questionnaires) | | Husbands’ use of empathic responding was associated with increased perception of same-day marital tension, whereas wives’ use was associated with decreased same-day marital tension. However, empathic responding decreased the subsequent day’s marital tension for both partners. | |
| RFM | Schokker, Sutive, Bouma, Keers, Links, Wolffenbuttel, Sanderman, & Hagedoorn (2010) | 205 Dutch couples w/ a partner w/ type 1 or type 2 diabetes | | WGS | | Longitudinal | | In both partners relationship satisfaction related positively to received active engagement and negatively to received protective buffering. Active engagement, when low, moderates the negative association between protective buffering and relationship satisfaction. | |
| RFM | Snippe, Maters, Wempe, Hagedoorn, & Sanderman (2012) | 68 Dutch couples w/ a partner w/ COPD | | WGS | | Cross-sectional | | When taken together, only protective buffering but not overprotection was associated with distress. Discrepancies between patients’ and partners’ perceptions of overprotection were associated with more distress of patients. | |
| RFM | Sormanti, Kayser, & Strainchamps (1997) | 34 US women with breast cancer | | RFCS | | Cross-sectional | | Higher protective buffering and lower active engagement were not significantly related with quality of life, depression or health-care behaviors. | |
| RFM | Suls, Green, Rose, Lounsbury, & Gordon (1997) | 43 US couples w/ men survivors of an acute myocardial infarction | | RFCS | | Cross-sectional | | Patients' and partners' protective buffering showed actor effects on distress cross-sectionally and for patients' also prospectively. No partner effects were found. | |
| RFM | Vilchinsky, Dekel, Leibowitz, Reges, Khaskia, & Mosseri (2011) | 86 Israelian couples w/ men w/ with first myocardial infarction or unstable angina | | WGS | | Longitudinal | | Female partners’ protective buffering was positively associated with male patients’ depressive symptoms at follow-up only when male patients’ own perceptions of partners’ protective buffering were low. Active engagement was associated with higher chances of smoking cessation. | |
| STM | (1) Austin & Falconier, 2013; (2) Falconier (2013); (3) Falconier, Nussbeck, & Bodenmann (2013a); (4) Falconier, Nussbeck, & Bodenmann (2013b) | 104 Latino immigrant couples in the U.S. | | | DCI-37 | Cross-sectional | | (1) Each partner’s spirituality had a direct positive effect on supportive and common DC. Common DC mediated the relation between spirituality and psychological aggression; (2) More traditionally gender role oriented Latino men were less likely to engage in supportive and common DC whereas Latinas’ levels of traditional gender role orientation had no effect on their DC; (3) Common and male supportive DC attenuate the negative association of their female partner's immigration stress; (4) All STM total DC and its dimensions associated with relationship satisfaction and most with individual engagement coping. | |
| STM | Badr, Carmack, Kashy, Cristofanilli, & Revenson (2010) | 191 US couples w/ women w/ cancer | | | DCI-55 adjusted to cancer-related stress | Longitudinal | | Common DC was related with higher distress in patients but better dyadic adjustment for both partners. Supportive DC was negatively related with distress for both partners. | |
| STM | Bergstraesser, Inglin, Hornung, & Landolt (2015) | 46 Swiss married parental couples | | | DCI-55 | Cross-sectional (interviews-questionnaires) | | DC played an important role in grief work and adjustment to bereavement, as aspects of common DC helped to work through grief individually and as a couple. | |
| STM/  RFM | Bertoni, Donato, Graffigna, Barello, & Parise (2015) | 100 Italian cardiac patients and 46 partners | | | DCI-18, Overprotection: The Michigan Family Heart Questionnaire (Fiske et al., 1991) | Cross-sectional | | Partners’ negative DC correlated positively with partner engagement and partners’ couple satisfaction correlated negatively with partner engagement. | |
| STM | Bodenmann & Cina (2005) | 62 Swiss couples | | | DCI-18 | Longitudinal | | Stable-satisfied couples reported engaging in more emotion-focused supportive and common DC and emotion focused CDC than distressed or separated/divorced couples. | |
| STM | Bodenmann & Perrez (1991) | 22 Swiss couples (12 in experimental, 10 in control) | | | Observational | Longitudinal, Pilot study, experimental | | When stressed, couples exhibit more DC (problem- and emotion-focused); unhappy couples rather rely on factual stress communication and less emotional exchange. | |
| STM | Bodenmann & Widmer (2000) | 242 Swiss couples | | | DCI-55 | Cross-sectional | | In the comparison of three age-groups, the oldest couples had the lowest values in positive DC and higher values in problem-oriented/factual stress communication; they are also less satisfied with the partner support. | |
| STM | (1) Bodenmann (1995); (2) Bodenmann (1997); (3) Bodenmann & Cina (1999), (4) Bodenmann & Cina (2000) | 70 Swiss couples | | | DCI-18 | Longitudinal, experimental (observational, questionnaires) | | DC acts as a moderator between stress and relationship outcomes, stable-satisfied couples indicated a lower level of stress and relied more frequently on DC, stress and DC both have high predictive values (e.g. positive DC accounts for 30% of the variance of relationship satisfaction), even in the long-term after 4 years. After five years, 73 3% of the couples could correctly be classified by using only two groups (stable versus separated/divorced). | |
| STM | Bodenmann (2000) | different Swiss samples | | | DCI-62 | Cross-sectional | | Especially emotional supportive and common DC are associated with relationship quality; couples with high stress but high DC are able to stay on a stable level during a 5-year period, whereas couples with high stress but low DC decline in relationship satisfaction. | |
| STM | Bodenmann, Atkins, Schär, & Poffet (2010) | 103 Swiss females | | | DCI-37 | Longitudinal | | DC was associated positively with orgasms, sexual behavior and sexual satisfaction. No moderating effects of DC or individual coping on the association between stress and sexuality. | |
| STM | Bodenmann, Charvoz, Widmer, & Bradbury (2004) | 106 depressed or formerly depressed Swiss individuals and 106 Swiss matched controls | | | DCI-55 | Cross-sectional | | Highly depressed women report less stress communication and own supportive DC compared to controls and partially remitted women. | |
| STM | Bodenmann, Cina, & Schwerzmann (2001) | 39 clinically depressed Swiss patients and 21 former depressed subjects | | | DCI-55 | Cross-sectional | | Severely depressed patients have a severe lack of positive DC resources; remitted patients did not differ from controls in their coping; coping deficiencies do not represent a stable personality trait. | |
| STM | Bodenmann, Meuwly, Bradbury, Gmelch, & Ledermann (2010) | 317 Swiss individuals | | | DCI-37 | Cross-sectional | | Both positive and negative forms of DC moderated the effects of stress on verbal aggression when levels of stress were low. At high levels of stress, neither form of DC attenuated the association between stress and verbal aggression. | |
| STM/ CM | Bodenmann, Meuwly, & Kayser (2011) | 443 Swiss couples | | | DCI-37  COPE | Cross-sectional | | Positive DC was a significant predictor of relationship quality and negative DC predicted quarrelling. DC was a weaker predictor of individual well-being. STM variables were stronger predictors of relationship quality and health than CCM variables. | |
| STM | Bodenmann, Meyer, Binz, & Brunner (2004) | 1783 Swiss married couples | | | DCI-55 | Cross-sectional | | The couple typology by Gottman (1993, 1994) was replicated; all three types of functional couples reported significantly higher marital satisfaction, volatile couples reported the highest scores in nearly all positive variables (e.g. DC). | |
| STM | Bodenmann, Pihet, & Kayser (2006) | 90 Swiss couples | | | DCI-55 | Longitudinal | | DC was positively associated with couples’ tenderness and togetherness and decreased quarrelling. Men’s supportive DC was more important for women than vice versa. Negative DC negatively predicts marital quality in both partners. | |
| STM | Donato, Iafrate, Barni, Bertoni, Bodenmann, & Gagliardi (2009) | 389 Italian couples | | | DCI-41 | Cross-sectional | | The DCI factorial structure was confirmed. The difference in supportive DC between emotion- and problem-focused was more relevant for women than for men. Common CD included three dimensions. | |
| STM | Donato, Iafrate, Bradbury, & Scabini (2012) | 153 Italian premarital couples, 128 women's parents and 101 men's parents | | | DCI-41 | Cross-sectional | | Parents and their children are similar in their negative DC but not in their positive DC. | |
| STM | Donato, Parise, Iafrate, Bertoni, Finkenauer, & Bodenmann (2015) | 114 Italian couples | | | DCI-41 | Longitudinal | | Partners' change in reported DC behaviors are reflected in their own and the other partner's change in perceptions. Men and women do not differ in either reported or perceived coping. DC reported behaviors predict relationship satisfaction partially through increases in own perceptions of the other's dyadic coping behaviors. | |
| STM | Feldman & Broussard (2006) | 71 US male partners of breast cancer patients | | | DCI-61 | Cross-sectional | | The less negative DC men reported, the better the physical well-being of their wives diagnosed with breast cancer. Men with a history of depression used more hostile and avoidant DC. | |
| STM | (1) Gabriel & Bodenmann (2006a); (2) Gabriel & Bodenmann (2006b) | 96 Swiss couples w/ a child | | | DCI-41 | Cross-sectional | | Parents of a child with inattention and conduct problems reported higher levels of stress and less competencies in DC and more child-related marital conflicts. | |
| STM | Gabriel, Untas, Layner, Koleck, & Luminet (2016) | 112 French couples | | | DCI-37 | Cross-sectional | | For women, association between anxiety/depression and DC was mediated by alexithymia but for men association between alexithymia and DC was mediated by anxiety/depression. | |
| STM | (1) Gabriel, Zeender, & Bodenmann (2005); (2) Gabriel, Zeender, & Bodenmann (2008) | 43 Swiss couples w/ a normally developed child, 75 couples w/ a child with Down syndrome, 54 parents w/ a child w/externalizing behavior problems | | | DCI-41 | Cross-sectional | | Parents of a child with Down syndrome reported more stress, but also high scores of DC whereas parents of a child with externalizing behavior problems perceived more stress and a lack of DC (more negative DC, less common DC). | |
| STM | Gagliardi, Bodenmann, Heinrichs, Bertoni, Iafrate, & Donato (2013) | 304 couples (Swiss, German, Italian) | | | DCI-37 | Cross-sectional | | Securely attached couples reported better relationship quality as well as more positive and less negative DC compared to fearful-avoidant couples and couples with different attachment styles. | |
| STM | Gagliardi, Bodenmann & Bregy (2010) | 225 Thai-Swiss couples and 234 Swiss couples | | | DCI-37 | Cross-sectional | | Bi-national couples showed less negative DC and stress communication than Swiss couples. | |
| STM | Gagliardi, Bodenmann, & Heinrichs (2015) | 154 Swiss and German couples | | | DCI-37 | Cross-sectional | | A passionate love style is most strongly associated with DC, whereas a rather rational love style was predictive for men's negative but women's positive DC. | |
| STM | García-López, Sarriá, Pozo, Recio (2016) | 67 Spanish parents of children w/ Autism Spectrum Disorder | | | DCI-5 | Cross-sectional | | Relationship satisfaction mediated the association between supportive DC and parental adaptation. | |
| STM | Gmelch & Bodenmann (2007) | 443 Swiss couples | | | DCI-37 | Cross-sectional | | Comparison of own and partner perspective of the DCI showed that discrepancy indexes and equity index were related to relationship quality; women’s appraisals of DC were slightly more relevant than the men’s appraisal of DC for relationship satisfaction. | |
| STM | Gmelch, Bodenmann, Meuwly, Ledermann, Steffen-Sozinova & Striegl (2008) | 2,399 Swiss heterosexual subjects | | | DCI-37 | Cross-sectional | | Results provide empirical evidence for the quality (internal consistency, test-retest correlation, validity, clinical application) of the DCI | |
| STM | Gouin, Scarcello, da Estrela, Paquin, & Barker (2016) | 44 Canadian parents of children w/ Autism Spectrum Disorder | | | DCI-37 | Cross-sectional | | Higher positive DC was significantly associated with fewer markers of systemic inflammation, while negative DC was unrelated to inflammation. | |
| STM | Herzberg (2013) | 240 German middle-aged couples | | | DCI-37 | Cross-sectional | | Perception of common DC positively associated with own relationship satisfaction. Compared to individual coping, emotion and problem-focused common DC found to be a stronger predictor of relationship satisfaction. | |
| STM | Hilpert et al. (2016) | 7973 married individuals from 35 nations | | | DCI-4 | Cross-sectional | | DC was a significant predictor of relationship satisfaction across all 35 nations, however the mean level of DC and relationship satisfaction as well as the association between the two variables differed between nations suggesting that the coping process in couples is influenced by culture. | |
| STM | Hilpert, Bodenmann, Nussbeck, & Bradbury (2013) | 1944 married Swiss individuals | | | DCI-3 | Cross-sectional | | External stress is highly associated with an increase in negative interactions and a decrease in DC; being supported by the partner in times of need seemed to be particularly relevant for marital quality. | |
| STM | Iafrate, Bertoni, Donato, & Finkenauer (2012) | 197 young premarital Italian couples and 192 Italian couples, parents of younger couples | | | DCI-41 | Cross-sectional | | Similarity of the own and the partner perception of own supportive DC was not related to relationship satisfaction in either young or older couples. | |
| STM | Iafrate, Bertoni, Margola, Cigoli, & Acitelli (2012) | 281 Italian couples | | | DCI-41 | Cross-sectional | | The relationship satisfaction of both partners was predicted by perceived but not actual similarity of DC (emotion- and problem-focused). For women, perceived similarity remained significant when adjusted for stereotype effects. | |
| STM | (1) Johnson & Horne (2016), (2) Johnson, Horne, & Galovan (2016) | 1,427 German couples | | | DCI-3 | Longitudinal | | (1) Participants’ and partners’ supportive DC predicted higher levels of commitment and willingness to sacrifice, and willingness to sacrifice predicted less supportive dyadic coping only for participants. No moderating effect of gender. (2) Associations among the variables. Higher levels of economic pressure and a greater number of children in the couple’s household were each associated with a steeper decline over 5 years in supportive DC. | |
| STM | Körner, Würner, Brosseau, Brähler, Kapellen, & Liess (2013) | 38 German parents of children w/ Type 1 diabetes | | | DCI-55 | Cross-sectional | | Better health indicators when parents reported more stress communication and dyadic coping. | |
| STM | Kramer, Ceschi, Van der Linden, & Bodenmann (2005) | 36 Swiss couples: 18 couples w/ a partner surviving a traumatic event and 18 control couples | | | DCI-55 | Cross-sectional | | People show less positive DC after traumatic events and engage more in negative DC compared to controls. Partners of the person that experienced the trauma seem less affected. | |
| STM | Lambert, Hasbun, Engh, & Holzer (2015) | 56 US civilian women and their combat veteran husbands | | | DCI-37 | Cross-sectional | | The degree to which a veteran’s PTSS was negatively associated with his spouse’s relationship quality depended on whether she perceived him as supportive when she experienced stress (i.e., supportive DC) and the degree to which she perceived the couple as working together to manage difficulties (i.e., common DC). | |
| STM | Landis et al. (2014) | 201 Swiss couples | | | DCI-37 | Cross-sectional | | Relationship satisfaction mediated the effects between commitment and common DC. | |
| STM | Landis, Peter-Wight, Martin, & Bodenmann (2013) | 132 Swiss and German elderly couples in long-term marriages | | | DCI-41 | Cross-sectional | | Partner’s perception of their spouse’s supportive DC was more strongly linked to their relationship satisfaction than to their own DC. The perception of DC was more important for marital satisfaction than coping congruency. | |
| STM | Ledermann et al. (2010) | 216 Swiss individuals, 378 Italian, and 198 French. | | | DCI-37 | Cross-sectional | | The factor structure of the original inventory was able to be replicated in all three language groups. DC was more strongly related to marital quality than to dyadic communication. Associations between delegated DC and marital communication were somewhat lower than for the other DC subscales. | |
| STM | Levesque, Lafontaine, Caron, & Fitzpatrick (2014) | 709 Canadian university students | | | DCI-37 | Cross-sectional | | Support for the DCI factorial structure, reliability, and concurrent and discriminant validity. All DCI subscales associated with relationship satisfaction and negative DC positively associated with length of relationship. | |
| STM | Levesque, Lafontaine, Caron, Flesch, & Bjornson (2014) | 187 Canadian couples | | | DCI-37 | Cross-sectional | | Propensity for dyadic empathy predicted own DC strategies, which in turn predicted own relationship satisfaction, with DC having a mediating role among men. Men’s perspective-taking predicts women’s DC and women’s empathic concern predicts men’s DC. | |
| STM | (1) Meier, Bodenmann, Mörgeli, & Jenewein (2011); (2) Meier, Bodenmann, Mörgeli, Peter-Wight, Martin, Büchi, & Jenewein (2012) | 43 Swiss couples w/ a partners w/ chronic obstructive pulmonary disease (COPD) and 138 married healthy couples | | | DCI-37 | Cross-sectional | | (1) A higher imbalance in delegated DC was associated with a lower quality of life; more negative and less positive DC were associated with lower quality of life and higher psychological distress in couples with COPD; (2) DC of couples with COPD is unbalanced and more negative compared to that of healthy couples. | |
| STM | Merz, Meuwly, Randall, & Bodenmann (2014) | 131 Swiss couples | | | DCI-37 | Longitudinal | | DC was found to decrease the impact of chronic external stress on chronic internal stress (spillover), particularly in women. | |
| STM | (1) Meuwly, Bodenmann, Germann, Bradbury, Ditzen, & Heinrichs (2012); (2) Kuhn, Milek, Meuwly, Bradbury & Bodenmann (2017) | (1) 123 Swiss couples w/ subsamples experimentally stressed, (2) 127 Swiss couples experimentally stressed | | | DCI-37 | Longitudinal, experimental | | (1) Stressed individuals recovered faster from stress the more positive DC they received from the partner, with women high in attachment anxiety benefiting less from these behaviors. More avoidant individuals did not differ from less avoidant individuals in the extent to which they benefited from partner’s positive support.  (2) Problem-oriented stress expression was strongly linked to problem-oriented dyadic coping in a time sequence of 10s within a conversation, while emotion-oriented stress expression was associated with emotion-oriented dyadic coping reactions. | |
| STM | Meuwly, Feinstein, Davila, Nuñez, & Bodenmann (2013) | 82 Swiss women w/ either a male or a female partner | | | DCI-37 | Cross-sectional | | Homosexual women reported receiving better DC from and experiencing less conflict with their female partners compared to heterosexual couple. | |
| STM | (1) Meyer, Bodenmann, Binz, & Brunner (2005); (2) Nussbeck, Hilpert, & Bodenmann (2012) | 2,583 Swiss individuals | | | (1) DCI-4, (2) DCI-6 | Cross-sectional | | (1) Middle-aged couples show the highest amounts of DC whereas older couples show the lowest scores. (2) DC can explain variance in relationship satisfaction above positive interaction. | |
| STM | Papp & Witt (2010) | 100 US heterosexual dating couples | | | DCI-37 | Cross-sectional | | Negative mood regulation positively associated with positive DC. Positive and negative DC are stronger predictors of relationship satisfaction than individual coping and seem to be more important for women's relationship satisfaction. | |
| STM | Randall, Hilpert, Jimenez-Arista, Walsh, & Bodenmann (2015) | 938 US individuals | | | DCI-37 | Cross-sectional | | The DCI factorial structure, construct validity, and measurement invariance across gender and culture (compared with Swiss sample) was supported. | |
| STM | Reed, O'Connor, Pace, Raison, & Butler (2016) | 24 US healthy couples | | | DCI-37 | Longitudinal, experimental | | Couples low in DC showed immune reactivity to the laboratory interpersonal stress but did not differ from couples high in DC on immune recovery. | |
| STM | Rottmann et al. (2015) | 538 Dutch couples w/ women w/ breast cancer | | | DCI-37 | Longitudinal | | Common DC was associated with higher relationship quality and fewer depressive symptoms for both partners. Patients experienced more depressive symptoms the more delegated DC they provided to the partner. Partners experienced more depressive symptoms the more supportive DC the patient provided to them. | |
| STM | Ruffieux, Nussbeck, & Bodenmann (2014) | 162 Swiss couples | | | DCI-55 | Longitudinal | | Men's relationship satisfaction could be predicted by their DC 10 years prior. | |
| STM | Rusu, Hilpert, Beach, Turliuc, & Bodenmann (2015) | 215 Romanian couples | | | DCI-37 | Cross-sectional | | Supportive DC mediates the association between sanctification of the marriage and marital satisfaction & well-being. | |
| STM | Rusu, Hilpert, & Bodenmann (2016) | 510 Romanian couples | | | DCI-37 | Cross-sectional | | Support for DCI factorial structure, validity, and reliability of Romanian DCI. Women reported higher stress communication and total dyadic coping than men. | |
| STM | Schär & Bodenmann (2011) | 60 Swiss couples w/ a depressed partner | | | DCI-37 | Longitudinal, experimental | | Positive DC can explain additional variance in the course of depressive symptoms during therapy compared to only intrapersonal variables. | |
| STM | Vaske, Thöne, Kühl, Keil, Schürmann, Rief, & Stenzel (2015) | 63 German couples w/ a partners w/ COPD | | | DCI-37 | Longitudinal | | Patients’ quality of life (QoL) at follow-up was positively influenced by partners’ stress communication, and partner's QoL was negatively influenced by negative DC and positively influenced by delegated DC. | |
| STM | Vedes, Nussbeck, Bodenmann, Lind, & Ferreira (2013) | 605 Portuguese individuals | | | DCI-37 | Cross-sectional | | DCI factorial structure and convergent and criterion validity supported. | |
| STM | Vedes, Hilpert, Nussbeck, Randall, Bodenmann, & Lind (2016) | 92 Swiss heterosexual couples | | | DCI-37 | Cross-sectional | | Supportive and common DC partially mediated the association between different love styles and relationship satisfaction. | |
| STM | Weißflog et al. (2016) | 330 German couples w/ a partner w/ a hematologic malignancy | | | DCI-37 | Cross-sectional | | Stress communication of patients' partners was lower compared to that of a non-cancer sample. Patients reported receiving more delegated DC from their partners. Negative DC was associated with higher unmet supportive care needs. | |
| STM | Witkovsky & Braakmann (2015) | 31 German and Austrian PTSD patients | | | DCI-37 | Cross-sectional | | Low discrepancies between partners’ DC were accompanied by high relationship satisfaction, independent of the symptom severity of PTSD. However, higher severity of PTSD was related to more dysfunctional coping. | |
| STM | Wunderer & Schneewind (2008) | 663 German first marriage couples | | | DCI-5 | Cross-sectional | | DC mediates the positive association between relationship standards and relationship satisfaction. | |
| STM | Xu, Hilpert, Randall, Li, & Bodenmann (2016) | 474 Chinese couples | | | DCI-37 | Cross-sectional | | Support for DCI factorial structure, validity, and reliability of Chinese DCI. Less positive but more negative DC than Swiss and American samples. Gender differences in stress communication. | |
| STM | Yokotani & Kurosawa (2015) | 44 Japanese couples | | | DCI-37 | Cross-sectional | | Stress communication and common DC associated with own marital satisfaction for both partners. Husbands’ supportive and delegated DC predicted own marital satisfaction only for husbands. Negative DC negatively associated with marital satisfaction only for wives. | |
| STM | Zeidner, Kloda, & Matthews (2013) | 100 Israelian newlywed couples | | | DCI-37 | Cross-sectional | | DC mediates the positive association between emotional intelligence and relationship satisfaction. | |
| STM | Zemp, Bodenmann, Backes, Sutter-Stickel, & Revenson (2016) | (1) 289 Swiss mothers and 65 Swiss fathers; (2) 81 Swiss boys and 106 Swiss girls aged 11-13; (3) 38 Swiss families | | | (1) and (2): DCI-10  (3) Observational | Cross-sectional | | (1) Parent’s DC was negatively related to children’s internalizing and externalizing symptoms and positively to children’s prosocial behavior. The child’s gender had a moderating effect only on prosocial behavior with greater effect on girls. (2) Children’s perspective on parents’ DC was related negatively to internalizing and externalizing symptoms and positively to prosocial behavior. No gender moderating effect. (3) Observed parents’ DC associated negatively with children’s externalizing behaviors only. No gender moderating effect. | |
| STM | Zimmermann, Scott, & Heinrichs (2009) | 98 German couples w/ women with early stage breast cancer | | | DCI-41 | Cross-sectional | | Female's relationship satisfaction and perception of common DC predicted women's perceptions of their partner's acceptance of their appearance. DC predicted better perceived partner acceptance. | |
| RCCM | Kayser, Watson, & Andrade (2007) | 10 US couples w/ women w/ non-metastatic breast cancer | | | Individual Interviews | Cross-sectional, Qualitative | | Couples that used mutually responsive DC often identified a strengthening of the relationship after stressor. Couples who used disengaged avoidance tended to appraise the breast cancer as an individual stressor which negatively influenced the relationship. | |
| RCCM | Kayser et al. (2014) | 28 couples from Hong Kong-China, India, and the US | | Interviews | | Cross-sectional, Qualitative | | Four factors affecting DC with breast cancer: family boundaries, gender roles, personal control, and interdependence. | |
| DCCM | (1) Berg et al. (2008); (2) Berg, Xu, Hilpert, Randall, Li, & Bodenmann Wiebe, & Butner (2011) | 59 US couples w/ men w/ prostate cancer | | Collaborative Coping: Perceptions of Collaboration (Berg et al., 2009) | | Longitudinal (daily diary) | | Collaborative coping is associated with positive emotions, coping effectiveness, and marital satisfaction for both partners at the same day. Collaborative coping exacerbates the negative affect covariation between the spouses for wives when spouses perceive the same stressful event. | |
| DCCM | Checton, Magsamen-Conrad, Venetis, Greene (2015) | 308 US couples w/ a partner w/ a chronic health condition | | support as a proxy for DC (based on Greene et al., 2012) | | Cross-sectional | | Illness interference was a stronger predictor (albeit negative) of patients’ health condition management than was partner support. The more partners perceived that the patient’s illness interfered in their lives, the less patient support they perceived. | |
| DCCM | Dagan, Sanderman, Shokker, & Bass (2011) | 70 Dutch couples w/ a partner colorectal cancer | | Two subscales of the Interaction of Social Support list (Kempen & Van Eijk, 1995) | | Longitudinal | | Perceived spousal supportive behavior was negatively associated with distress 6 months later but only for patients relatively low in perceived personal control. Couples with a high sense of personal control reported lower levels of distress, regardless of partner support. | |
| DCCM | Lyons, Miller, & McCarthy (2016) | 78 US couples living with non-small cell lung cancer | | Emotional-Intimacy Disruptive Behavior scale (Druley, Stephens, & Coyne, 1997) | | Cross-sectional | | Controlling for developmental and contextual covariates, patients that engaged in higher levels of protective buffering were more likely to have worse mental health. | |
| DCCM | Magsamen-Conrad, Checton, Venetis, & Greene (2015) | 83 US couples w/ a partner w/ cancer | | Ability to communicate about cancer (communication efficacy; Checton et al., 2012) | | Cross-sectional | | Proximal contextual factors in form of relationship quality are related to dyadic adjustment (cancer management) and dyadic coping (communication efficacy). Perceptions of providing support and receiving support associated with relationship quality. | |
| DCCM | Schindler, Berg, Butler, Fortenberry, & Wiebe (2010) | 61 US couples w/ a partner w/ prostate cancer | | Perceptions of Collaboration Questionnaire (Berg, Schindler, & Maharajh, 2008) | | Cross-sectional | | The more partners enjoyed collaborative coping, the more common goals (shared selves) and well-being they reported regardless of age, marital quality, and subjective health. | |
| DCCM | Watts, Sherman, Mireskandari, Meiser, Taylor, & Tucker (2011) | 94 Australian couples w/women at increased risk for breast/ovarian cancer | | perception of support, satisfaction with support, and collaborative coping (self-developed) | | Cross-sectional | | Collaborative coping was associated positively with couple’s cohesion and partner’s support associated positively with couple’s consensus. These two forms of dyadic coping were rated similarly by the two partners and were associated with relationship satisfaction. | |
| CMM | Hallgren & McCrady (2015) | 188 American couples in couple behavioral alcohol treatment | | R script (Pennebaker, Francis, & Booth, 2001) of first and mid-treatment sessions | | Longitudinal | | Greater use of *we* language by patients and partners during both sessions was correlated with more improvement in abstinent days during treatment and abstinence at follow-up. Greater use of *we* language and lower use of *you* language by patients were the best predictors of improvements in abstinence during treatment. Communal coping predicts better abstinence outcomes in couple alcohol treatment. | |
| CMM | Lin, Chen, & Li (2016) | 31 Taiwanese dual-earner couples | | LIWC (Interviews) | | Longitudinal | | *We*-talk by wives was associated with increased work and marital satisfaction in husbands However, *we*-talk by husbands decreased wives’ work satisfaction. | |
| CMM | Robbins, Mehl, Smith, & Weihs (2013) | 75 American women w/ breast cancer, their partners, and one child | | LIWC (Interviews) | | Cross-sectional | | Partners’ use of *we*-talk related to patient’s lower depression and couple’s better couples adjustment. | |
| CMM | Rogers et al. (2016) | 20 Kenyan HIV positive pregnant women and 20 Kenyan HIV negative and their HIV positive or negative male partners | | Interviews | | Cross-sectional, Qualitative | | Communal coping (mutual disclosure, couples testing and counseling, and assistance with medications and appointments) helped HIV-negative couples try to avoid HIV acquisition and helped serodiscordant couples prevent HIV transmission and lived positively with HIV. | |
| CMM | Rohrbaugh, Mehl, Shoham, Reilly, & Gordon (2008) | 60 American patients w/ heart failure and their spouses | | LIWC (interviews) Two self-report questions | | Longitudinal, Qualitative | | *We* talk by the spouse, but not the patient, predicted positive change in the patient’s symptoms and general health over the next 6 months and did so better than direct self-report measures of marital quality and the communal coping construct. *We* talk by the patient and spouse did not correlate. No moderating gender effect on *we* talk. | |
| CMM | Rohrbaugh, Shoham, Skoyen, Jensen, & Mehl (2012) | 20 American couples w/ one partner continuing to smoke despite heart or lung disease in smoking cessation program | | LIWC (Interviews) | | Longitudinal, Qualitative | | Pre-treatment we-talk by the patient’s spouse predicted the patient’s abstinence 12 months after quitting, and residualized change in *we*-talk by both partners during the course of intervention (controlling for baseline levels) predicted cessation outcomes as well. | |
| CMM | Stachowski & Stephenson (2015) | 447 American gay/bisexual men and their partners | | HIV Communal Coping Strategies (Salazar, Stephenson, Sullivan, & Tarver, 2013) | | Cross-sectional | | Internalized homophobia was associated with greater communal coping whereas external homophobic discrimination was not. | |
| *Note.* WGS: Ways of Giving Support (Buunk et al., 1996); RFCS: Relationship-Focused Coping Scale (Coyne & Smith, 1991); WCCL: revised version of Ways of Coping Checklist (Vitaliano et al., 1985); LIWC: Linguistic Inquiry and Word Count system (Pennebaker, Francis, & Booth, 2001). Studies with same dataset are in the same cell and marked with (1) and (2), for example. | | | | | | | | | |
